# The efficacy and safety of mecobalamin combined with Chinese medicine injections in the treatment of diabetic peripheral neuropathy: A systematic review and Bayesian network meta-analysis of randomized controlled trials

**DOI:** 10.3389/fphar.2022.957483

**Published:** 2022-11-04

**Authors:** Yuqi Ma, Ji Chen, Xinggui Huang, Yuan Liu

**Affiliations:** School of Basic Medical Sciences, Chengdu University of Traditional Chinese Medicine, Chengdu, China

**Keywords:** Chinese medicine injections, diabetic peripheral neuropathy, network meta-analysis, efficacy, mecobalamin

## Abstract

**Background:** In recent years, people pay more and more attention to diabetic peripheral neuropathy (DPN). As a neurotrophic agent, mecobalamin is able to repaire nerves, which has already become a consensus among experts. However, it has been found that mecobalamin has poor effect to increase nerve conduction velocity, which is an important indicator. Clinical data have shown that Chinese medicine injection, combined with mecobalamin injection, can significantly improve nerve conduction velocity of the limbs. Nevertheless, several kinds of Chinese medicine injections have been used to treat DPN. The effect of these Chinese medicine injections for DPN are various. Therefore, it is necessary to evaluate the effectiveness of Chinese medicine injections combined with mecobalamin in the treatment of DPN.

**Methods:** All relevant articles published before 12 March 2022 were searched in eight electronic databases. Randomized controlled trials (RCTs) on Chinese medicine injections plus Mecobalamin for DPN were identified according to inclusion criteria, and were assessed using the revised Cochrane risk of bias tool (ROB2.0). R software and stata15 was used to create the ranking probabilities and network meta-analysis.

**Results:** A total of 80 RCTs involving 6,980 patients were included. The results showed that mecobalamin plus Dengzhanxixin injection (ME + DZXX) ranked first in overall response rate [RR = 1.64, 95% CI (1.26, 2.21)] and median motor nerve conduction velocity [MD = 9.46, 95% CI (5.67, 13.28)]. Then, mecobalamin plus Kudiezi Injection (ME + KDZ) had the best effect in median sensory nerve conduction velocity [MD = 10.41, 95% CI (−13.31, −7.52)], and mecobalamin plus Honghua injection (ME + HH) ranked highest in common peroneal motor nerve conduction velocity [MD = 6.8, 95% CI (4.13, 9.49)] and common peroneal sensory nerve conduction velocity [MD = −6.25, 95% CI (−8.85, −3.65)].

**Conclusion:** This study determined the efficacy of different Chinese medicine injections combined with mecobalamin. DZXX may be the best adjunctive Chinese medicine injection for DPN patients. However, due to potential risk of bias and limited RCTs, our results need to be treated with reservations.

## 1 Introduction

Diabetic peripheral neuropathy (DPN) is one of the common complications seen in patients with diabetes, and also the main cause of disability and death of diabetes. The clinical symptoms are mainly numbness of the limbs, abnormal sensation, weakening or disappearance of tendon reflexes, etc. In serious cases, gangrene, ulcer, and even amputation may occur (Gong, 2020). With the aging population and unhealthy lifestyle, the number of diabetes patients is increasing more and more. According to current studies, about 10%–50% of people with diabetes may develop DPN, which is a serious medical issue (Aszmann et al., 2004). Currently, the main therapeutic strategies for DPN focus on glycemic control and symptom relief (Hemmingsen et al., 2013).

In 2021, all experts from the diabetes branch of the Chinese Medical Association agreed that mecobalamin injection could alleviate the symptoms related to DPN, and promoted the regeneration of the limbs’ nerves (Neurological Complications Group of Diabetes Branch of Chinese Medical Association LS Zhu and Wu, 2021). However, a study showed that although mecobalamin injection alone could improve the overall treatment efficacy, mecobalamin injection was not effective to increase nerve conduction velocity, which was a crucial indicator (Sawangjit et al., 2020).

Chinese medicine injection has been widely used to relieve DPN in clinical practice recently (Wang, 2022). According to TCM theory, DPN is categorized as “arthralgia syndrome” (Chongze Chen, 2012), Chinese medicine injections can activate blood circulation and remove blood stasis. Various Chinese medicine injections in combination with mecobalamin have been used to alleviate DPN and have increased nerve conduction velocity (Xiao, 2010; Chunhua Wang and Zhang, 2012a; Ye, 2012; Hui Sun, 2013; Shaoxiong Cai et al., 2013; Genli Han and Wang, 2013; Xuemei Dai, 2013; Wenle Sui, 2014; Wang, 2015a; Xiangxiang Zhou, 2015a; Yang et al., 2016; Zhang, 2016; Lirong Dong et al., 2018; Zhao, 2019; Zhao et al., 2021). Several Chinese medicine injections have been developed.

The purpose of this study is to assess the effectiveness of nine Chinese medicine injections combined with mecobalamin in the treatment of DPN.

## 2 Materials and methods

The protocol has been registered in PROSPERO (CRD42022316703). This study was reported in strict accordance with the standard format and meta-analysis specifications of the Preferred Reporting Items for Systematic Reviews and Meta-Analysis (the PRISMA NMA) (Hutton et al., 2015).

### 2.1 Literature retrieval

We searched eight databases including China National Knowledge Infrastructure (CNKI), Wanfang Database, Database of Chinese Sci-tech Periodicals (VIP), Chinese Biomedical Literature Database (CBM), PubMed, Cochrane Library, Embase, and Web of Science from inception to 12 March 2022. Both MeSH terms and free words were combined to retrieve relevant RCTs. The search strategies are shown in the Supplementary Material S1.

### 2.2 Inclusion criteria

#### 2.2.1 Types of studies

We included RCTs, published in English or Chinese, which investigating the effect of Chinese medicine injection combined with mecobalamin for patients with DPN.

#### 2.2.2 Types of participants

The participants were diagnosed according to the “Chinese Guideline for the Prevention and Treatment of Diabetes” (2010 Edition) (CD, 2012); The participants had limb sensory and motor neuropathy manifestations, such as: limb numbness, glove sensation, chills, hyperalgesia or decreased perception of pain and temperature; The participants had evidently weakened or even disappearance of patellar (knee tendon) reflex and ankle jerk (Achilles tendon) reflex during neurological examination; The results of electromyography suggested a slow-down of nerve conduction velocity; The participants aged 40–85 years old, and the treatment period lasted 2–4 weeks, regardless of gender and country.

#### 2.2.3 Interventions and comparison

The intervention group which adopted Chinese medicine injections combined with mecobalamin injection, while the control group used only mecobalamin injection.

#### 2.2.4 Outcome

The primary outcome was overall response rate and secondary outcomes included median motor nerve conduction velocity, median sensory nerve conduction velocity, common peroneal motor nerve conduction velocity, common peroneal sensory nerve conduction velocity, and adverse reactions.

### 2.3 Exclusion criteria

The exclusion criteria were as follows: 1) duplication; 2) no mecobalamin injection; 3) no relevant data.

### 2.4 Study selection

EndnoteX9 was used to manage all articles. After removing duplicates, two reviewers (YQM and XGH) scrutinized articles based on the eligible criteria. Then, the full text was read for screening. Disagreements were settled through team discussion or consultation with the third reviewer (YL).

### 2.5 Data extraction

Two reviewers (YQM and JC) extracted information with pre-designed extraction form. The extracted data included author names, publication dates, interventions (Chinese medicine injections and mecobalamin injections), treatment duration and outcome indicators (primary and secondary outcomes). The extracted data was cross-checked by two reviewers. Discrepancies between the two researchers in the process of study selection were resolved by consensus or negotiation with a third researcher (YL).

### 2.6 Assessment of risk of bias

The risk of bias of included studies was evaluated using the Cochrane risk of bias tool 2.0 (Rob 2.0) (Sterne et al., 2019). The Rob 2.0 assesses the risk of bias from five domains, including bias generated in the random process, bias deviating from the established intervention, bias of missing outcome data, bias of outcome measurement and bias of selective reporting of results. Two independent reviewers (YQM and JC) conducted evaluation of Rob 2.0 and any discrepancy was arbitrated by a third reviewer (YL).

### 2.7 Statistical analysis

Stata 15.0, R software and Microsoft Excel 2019 were adopted for statistical analysis. R software (version 4.2.0) was used for data synthesis. The mean difference (MD) and the 95% confidence interval (CI) of continuous variables were measured, and the relative risk (RR) of the 95% CI of dichotomous data was calculated. For dichotomous variables, relative risk (RR) was used as an effect size indicator with a confidence interval of 95% (95% CI) based on the overall response rate of patients (Dias et al., 2013; Mills et al., 2013). For continuous variables such as median motor nerve conduction velocity, median sensory nerve conduction velocity, common peroneal motor nerve conduction velocity and common peroneal sensory nerve conduction velocity, mean difference (MD) with 95% CI was calculated. The Chinese medicine injections were compared using the surface under the ranking plot (SUCRA). The SUCRA curves indicate the most effective and least effective treatments in percentages of 100% and 0%, respectively. SUCRA curves and consistency test were performed using Stata 15 software (Rücker and Schwarzer, 2015; Trinquart et al., 2016). The funnel plot was drawn and compared to determine whether publication bias existed in this network meta-analysis.

## 3 Results

### 3.1 Search results

**FIGURE 1:**
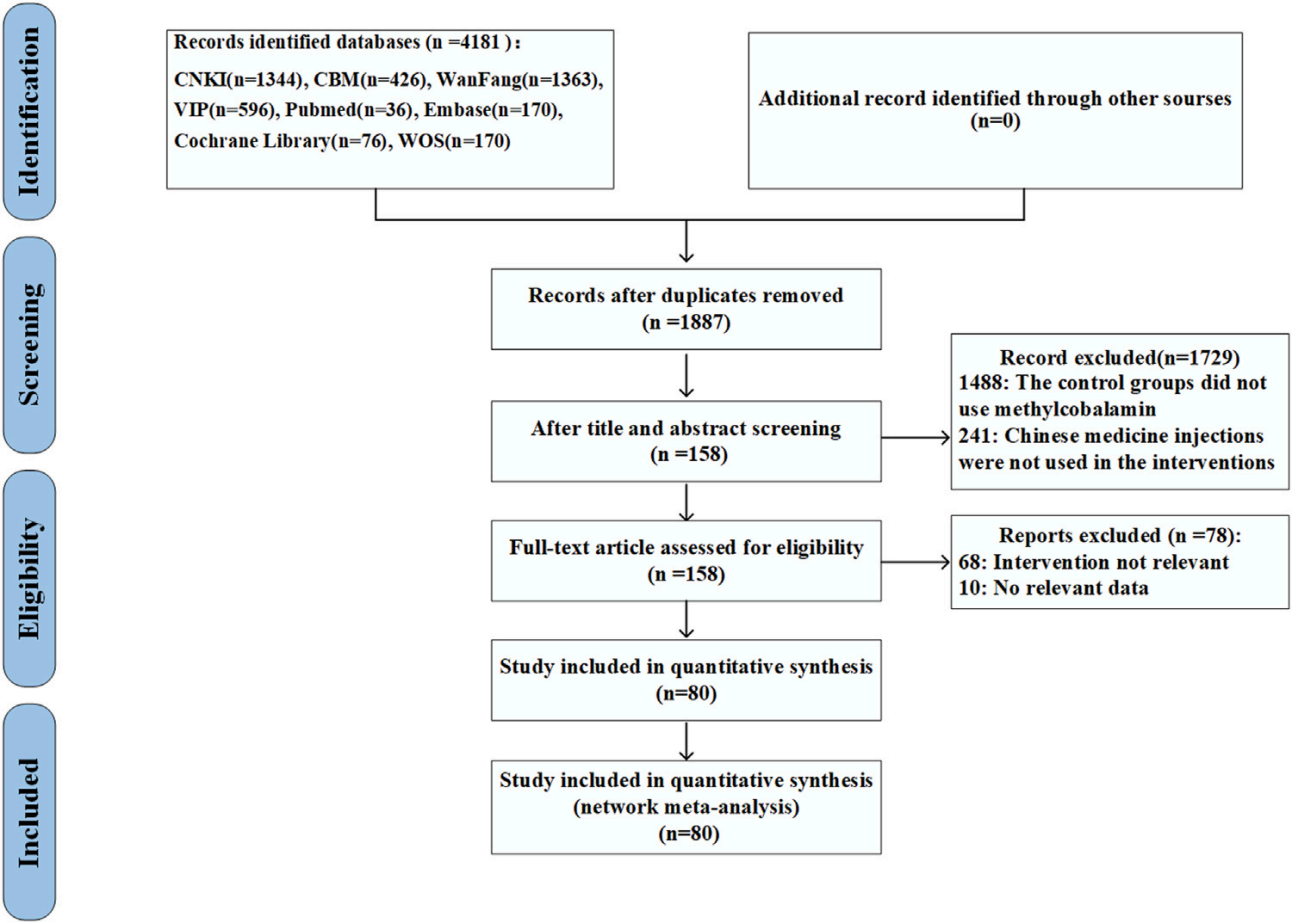
Flowchart of searching and screening for the studies.

A total of 4181 publications were searched initially, but only 1187 studies left after duplicates were deleted. The titles and abstracts were screened and 158 articles were selected for full-text assessment. Eighty trials were finally included in the present study based on eligible criteria. The detailed literature search process is shown in Figure 1.

### 3.2 The characteristics of included studies

The present study included a total sample size of 6980 cases, involving 9 interventions: mecobalamin injection + Dengzhan xixin injection (ME + DZXX) (Hong Li and Liang, 2009), mecobalamin injection + Honghua injection (ME + HH) (Huang, 2006; Wen, 2007; Dejian Liang and Zhu, 2010), mecobalamin injection + Danshenchuanxiongqin injection (ME + DSCXQ) (Peng, 2011; Tianjiang Li, 2011; XIan Xie et al., 2011; Hui Sun, 2013; Zhang, 2016; Lirong Dong et al., 2018; Zhao et al., 2021), mecobalamin injection + Yinxingye injection (ME + YXY) (Jiang, 2009; Fen Wang, 2009; Li, 2010a; Bao, 2010; Gao and Zhang, 2010; Xiao, 2010; Wunong Chen and Li, 2011; Yang, 2011; Chen, 2012; Fangye and Liu, 2012; Wang, 2012; Shaoxiong Cai et al., 2013; Xuemei Dai, 2013; Zhou, 2013; He Lu, 2015; Ying Li, 2017; Weiping Cao, 2019; Zhao, 2019), mecobalamin injection + Dengzhanhua injection (ME + DZHS) (Haiping Lan, 2007; Li, 2010b; Peng et al., 2012; Liao, 2014; Li, 2015), mecobalamin injection + Danhong injection (ME + DH) (Guo, 2008; Li, 2009; Shufen Sun, 2009; Wang et al., 2009; Yang, 2009; Yong Li, 2009; Li, 2010b; Guo, 2010; Hongli Cai et al., 2010; Ren, 2011; Wu, 2011; Zhang and Yue, 2011; Chunhua Wang and Zhang, 2012b; Deng, 2012; Liang Lv et al., 2012; Shao, 2012; Ye, 2012; Yong Yang et al., 2012; Zhilin Wang, 2012; Feng, 2013; Genli Han and Wang, 2013; Hu, 2013; Jinli Chen and Li, 2013; An, 2014; Fang He et al., 2014; Lei Xu and Chang, 2014; Li, 2014; Wenle Sui, 2014; Xia Wang and Zhu, 2014; Wang, 2015b; Xiangxiang Zhou, 2015b; Wang, 2015a; Wenjuan Qiu and Ding, 2015; Jin Xie et al., 2016; Yang et al., 2016; Hu, 2017), mecobalamin injection + Gegensu injection (ME + GGS) (Si, 2003; Zhang et al., 2007; Heying Li, 2011; Menghua Yang and Liu, 2022), mecobalamin injection + Chuanxiongqin injection (ME + CXQ) (Sun et al., 2007; Yu, 2008; Wang, 2009) and mecobalamin injection + Kudiezi Injection (ME + KDZ) (Shichao Teng and Wang, 2008; Peng et al., 2011; Fang Wang, 2020). The characteristics of included studies are shown in Table 1. The Supplementary Material S2 presents the specific information of all included Chinese medicine injections. The Chinese medicine injections’ details were showed in the Supplementary Materials S3, 4.

**TABLE 1:** Characteristics of included RCTs.

Studies Id	Treatment	Sample size	Age(Mean ± SD)	Gender (M/F)	Treatment duration	Outcomes
Si. (2003)	ME (0.5 mg/d+250 mlNS) + GGS (400 mg/d)	38	59.71 ± 14.27	20/18	4 weeks	a,b,c,d,e
Si. (2003)	ME (0.5 mg/d)	36	56.69 ± 7.11	19/17	4 weeks	a,b,c,d,e
Huang. (2006)	ME (500 μg/d) + HH(20 ml + 500 mlNS)	44	60.2 ± 4.8	18/26	4 weeks	a,b,c,d,e
Huang. (2006)	ME (500 μg/d)	44	64.1 ± 1.65	20/24	4 weeks	a,b,c,d,e
HaiPing Lan. (2007)	ME (150 mg/d) + DZHS(150 mg/d+250 mlNS)	43	53.2 ± 3.7	20/23	4 weeks	a,d,e
HaiPing Lan. (2007)	ME (150 mg/d)	39	53.6 ± 3.6	19/20	4 weeks	a,d,e
Sun et al. (2007)	ME (1500 μg/d) + CXQ (0.24 g/d)	63	58.94 ± 7.02	31/32	2 weeks	a,b,c,d,e
Sun et al. (2007)	ME (1500 μg/d)	63	59.03 ± 6.57	32/31	2 weeks	a,b,c,d,e
Zhang et al. (2007)	ME (500 μg/d) + GGS (0.4 g/d+250 mlNS)	30	62 ± 6.2	12/18	4 weeks	a,b,c,d,e
Zhang et al. (2007)	ME (500 μg/d)	30	60 ± 6.1	14/16	4 weeks	a,b,c,d,e
Wen, (2007)	ME (1000 μg/d) + HH(40 mg/d)	27	—	12/15	2 weeks	a,b,c,d,e
Wen, (2007)	ME (1000 μg/d)	25	—	13/12	2 weeks	a,b,c,d,e
ChangXiu Guo and Sun. (2008)	ME (500 μg/d) + DH (20 ml/d+250 mlNS)	38	49.5 ± 5.2	20/18	2 weeks	a,b,c,d,e
ChangXiu Guo and Sun. (2008)	ME (500 μg/d)	34	44.6 ± 4.2	18/16	2 weeks	a,b,c,d,e
Shichao Teng and Wang. (2008)	ME (0.5 mg/d+100 ml) + KDZ (30 ml/d+250 mlNS)	37	59.3 ± 12.3	26/11	2 weeks	a,b,c,d,e
Shichao Teng and Wang. (2008)	ME (0.5 mg/d+100 ml)	40	58.1 ± 11.8	29/11	2 weeks	a,b,c,d,e
Yu. (2008)	ME (500 μg/d) + CXQ (200 mg/d+250 mlNS)	86	38–76	47/41	2 weeks	a
Yu. (2008)	ME (500 μg/d)	82	40–78	50/32	2 weeks	a
Jiang (2009)	ME (0.5 mg/d) + YXY (20 ml/d+250 mlNS)	30	—	19/11	4 weeks	a
Jiang (2009)	ME (0.5 mg/d)	30	—	20/10	4 weeks	a
Li. (2009)	ME (0.5 mg/d) + DZXX (20 ml/d+250 mlNS)	64	60 ± 6.52	38/26	4 weeks	a,b,c,d,e
Li. (2009)	ME (0.5 mg/d)	56	60.5 ± 8.33	32/24	4 weeks	a,b,c,d,e
Li. (2009)	ME (0.5 mg/d) + DH (30 ml/d+250 mlNS)	34	59.2 ± 4.8	18/16	4 weeks	a,b,c,d,e
Li. (2009)	ME (0.5 mg/d)	33	60.2 ± 5.1	17/16	4 weeks	a,b,c,d,e
Yong Li. (2009)	ME (1 mg/d) + DH (20 ml/d+250 mlNS)	42	51.42 ± 6.41	20/22	2 weeks	a,b,c,d,e
Yong Li. (2009)	ME (1 mg/d)	42	51.8 ± 5.57	17/23	2 weeks	a,b,c,d,e
ShuFen Sun. (2009)	ME (0.5 mg/d) + DH (20 ml/d+250 mlNS)	52	51.2 ± 6.41	24/28	2 weeks	a
ShuFen Sun. (2009)	ME (0.5 mg/d)	50	51.8 ± 5.57	24/26	2 weeks	a
Fen Wang. (2009)	ME (0.5 mg/d+250 mlNS)+YXY (20 ml/d+250 mlNS)	46	42–74	25/21	3 weeks	a
Fen Wang. (2009)	ME (0.5 mg/d+250 mlNS)	40	40–72	22/18	3 weeks	a
Wang. (2009)	ME (0.5 mg/d) + DH (30 ml/d+250 mlNS)	30	52.4 ± 6.5	18/12	4 weeks	a,b,c,d,e
Wang. (2009)	ME (0.5 mg/d)	30	53.8 ± 5.7	17/13	4 weeks	a,b,c,d,e
Wang. (2009)	ME (0.5 mg/d+100 mlNS)+CXQ (80 ml/d+250 mlNS)	40	46–72	—	4 weeks	a
Wang. (2009)	ME (0.5 mg/d+100 mlNS)	36	46–72	—	4 weeks	a
Yang. (2009)	ME (0.5 mg/d) + DH (20 ml/d+200 mlNS)	33	—	15/18	4 weeks	a,b,c
Yang. (2009)	ME (0.5 mg/d)	34	—	16/18	4 weeks	a,b,c
Bao. (2010)	ME (0.5 mg/d+250 mlNS) + YXY (30 ml/d+250 mlNS)	30	60.2 ± 3.8	16/14	4 weeks	a
Bao. (2010)	ME (0.5 mg/d+250 mlNS)	30	59.4 ± 4.1	13/17	4 weeks	a
Hongli Cai. (2010)	ME (2 mg/d+100 mlNS)+DH (40 ml/d+250 mlNS)	50	65–95	27/23	4 weeks	a,b,c,d,e
Hongli Cai. (2010)	ME (2 mg/d+100 mlNS)	50	65–91	26/24	4 weeks	a,b,c,d,e
Gao and Zhang. (2010)	ME (0.5 mg/d) + YXY (20 ml/d+250 mlNS)	30	41–78	17/13	4 weeks	b,c,d,e
Gao and Zhang. (2010)	ME (0.5 mg/d)	30	42–81	18/12	4 weeks	b,c,d,e
Guo. (2010)	ME (0.5 mg/d+150 mlNS) + DH (40 ml/d+250 mlNS)	32	—	17/15	3 weeks	a
Guo. (2010)	ME (0.5 mg/d+150 mlNS)	32	—	15/17	3 weeks	a
Li. (2010a)	ME (0.5 mg/d) + DZHS(20 ml/d+250 mlNS)	35	57 ± 10	20/15	4 weeks	a,b,c,d,e
Li. (2010a)	ME (0.5 mg/d)	35	56 ± 10	19/16	4 weeks	a,b,c,d,e
Li (2010b)	ME (0.5 mg/d+100 mlNS)+YXY (20 ml/d+250 mlNS)	30	59 ± 11	18/12	4 weeks	a
Li (2010b)	ME (0.5 mg/d+100 mlNS)	30	60 ± 12	20/10	4 weeks	a
Li (2010b)	ME (0.5 mg/d) + DH (20 ml/d)	59	50 ± 8	—	2 weeks	a,b,c,d,e
Li (2010b)	ME (0.5 mg/d)	55	50 ± 8	—	2 weeks	a,b,c,d,e
DeJian Liang and Zhu. (2010)	ME (0.5 mg/d) + HH(20 ml/d+500 mlNS)	32	58 ± 10	16/16	4 weeks	a,b,c,d,e
DeJian Liang and Zhu. (2010)	ME (0.5 mg/d)	32	58 ± 10	18/14	4 weeks	a,b,c,d,e
Xiao. (2010)	ME (1.5 mg/d) + YXY (20 ml/d+250 mlNS)	38	—	21/17	2 weeks	a,b,c,d,e
Xiao. (2010)	ME (1.5 mg/d)	37	—	19/18	2 weeks	a,b,c,d,e
Wunong Chen and Li. (2011)	ME (0.5 mg/d) + YXY (20 ml/d+250 mlNS)	21	40–77	11/10	4 weeks	a,d,e
Wunong Chen and Li. (2011)	ME (0.5 mg/d)	21	47–76	9/12	4 weeks	a,d,e
Heying Li. (2011)	ME (0.5 mg/d) + GGS (300 ml/d+250 mlNS)	48	47 ± 11	23/25	6 weeks	a,d,e
Heying Li. (2011)	ME (0.5 mg/d)	46	47 ± 11.2	24/22	6 weeks	a,d,e
TianJiang Li. (2011)	ME (0.5 mg/d) + DSCXQ (10 ml/d+250 mlNS)	50	53–82	32/18	4 weeks	a,b,c,d,e
TianJiang Li. (2011)	ME (0.5 mg/d)	50	56–78	27/23	4 weeks	a,b,c,d,e
Peng. (2011)	ME (0.5 mg/d) + DSCXQ (20 ml/d+250 mlNS)	56	61.3 ± 8.6	31/25	2 weeks	a,d,e
Peng. (2011)	ME (0.5 mg/d)	56	62.9 ± 8.9	29/27	2 weeks	a,d,e
Peng et al. (2011)	ME (1 mg/d) + KDZ (30 ml/d+100 mlNS)	50	62.36 ± 12.39	26/24	3 weeks	a,b,c,d,e
Peng et al. (2011)	ME (1 mg/d)	48	64.36 ± 7.2	28/20	3 weeks	a,b,c,d,e
Ren. (2011)	ME (0.5 mg/d+100 mlNS) + DH (30 ml/d+250 mlNS)	33	—	—	2 weeks	a
Ren. (2011)	ME (0.5 mg/d+100 mlNS)	32	—	—	2 weeks	a
Wu. (2011)	ME (0.5 mg/d) + DH (20 ml/d+250 mlNS)	60	—	—	2 weeks	a,d,e
Wu. (2011)	ME (0.5 mg/d)	60	—	—	2 weeks	a,d,e
Xian Xie et al. (2011)	ME (0.5 mg/d+100 mlNS) + DSCXQ (15 ml/d+250 mlNS)	42	—	—	2 weeks	a,b,c,d,e
Xian Xie et al. (2011)	ME (0.5 mg/d+100 mlNS)	42	—	—	2 weeks	a,b,c,d,e
Yang. (2011)	ME (0.5 mg/d+250 mlNS)+YXY (20 ml/d+250 mlNS)	42	—	—	20days	a,b,c,d,e
Yang. (2011)	ME (0.5 mg/d+250 mlNS)	42	—	—	20days	a,b,c,d,e
Zhang and Yue. (2011)	ME (1 mg/d+100 mlNS) + DH (30 ml/d+250 mlNS)	24	51.7 ± 8	13/11	2 weeks	a
Zhang and Yue. (2011)	ME (1 mg/d+100 mlNS)	22	50.8 ± 9	12/10	2 weeks	a
Chen. (2012)	ME (0.5 mg/d) + YXY (20 ml/d+250 mlNS)	40	52.3	22/18	4 weeks	a,b,c,d,e
Chen. (2012)	ME (0.5 mg/d)	40	52.3	19/21	4 weeks	a,b,c,d,e
Deng. (2012)	ME (0.5 mg/d)+DH (20 ml/d+250 mlNS)	20	61.4 ± 9.5	14/6	2 weeks	a
Deng. (2012)	ME (0.5 mg/d)	20	61.8 ± 9.1	14/6	2 weeks	a
Liang Lv et al. (2012)	ME (1 mg/d+20 mlNS) + DH (40 ml/d+100 mlNS)	20	56.4 ± 16.9	13/17	2 weeks	a
Liang Lv et al. (2012)	ME (1 mg/d+20 mlNS)	20	50.6 ± 9.1	11/19	2 weeks	a
Peng et al. (2012)	ME (1 mg/d) + DZHS(75 mg/d+100 mlNS)	48	61.56 ± 12.18	28/20	2 weeks	a,b,c,d,e
Peng et al. (2012)	ME (1 mg/d)	40	62.36 ± 6.25	18/22	2 weeks	a,b,c,d,e
Shao. (2012)	ME (0.5 mg/d) + DH (20 ml/d+250 mlNS)	38	43.6 ± 15.4	21/17	4 weeks	a
Shao. (2012)	ME (0.5 mg/d)	30	45.1 ± 17.2	22/8	4 weeks	a
ChunHua Wang and Zhang. (2012b)	ME (1 mg/d+250 mlNS) + DH (20 ml/d+250 mlNS)	30	—	18/12	4 weeks	a,d,e
ChunHua Wang and Zhang. (2012b)	ME (1 mg/d+250 mlNS)	30	—	17/13	4 weeks	a,d,e
Wang. (2012)	ME (0.5 mg/d) + YXY (30 ml/d+250 mlNS)	46	52.8 ± 6.5	26/20	4 weeks	a,d,e
Wang. (2012)	ME (0.5 mg/d)	46	54.1 ± 6.4	24/22	4 weeks	a,d,e
Zhilin Wang. (2012)	ME (1 mg/d) + DH (20 ml/d+250 mlNS)	100	67.99 ± 8.63	61/39	4 weeks	a
Zhilin Wang. (2012)	ME (1 mg/d)	100	68.14 ± 8.96	64/36	4 weeks	a
Yong Yang et al. (2012)	ME (0.5 mg/d) + DH (20 ml/d+250 mlNS)	30	—	—	2 weeks	b,c,d,e
Yong Yang et al. (2012)	ME (0.5 mg/d)	30	—	—	2 weeks	b,c,d,e
Ye. (2012)	ME (0.5 mg/d) + YXY (20 ml/d+250 mlNS)	40	61.3 ± 5.4	23/17	2 weeks	a
Ye. (2012)	ME (0.5 mg/d)	40	59.5 ± 6.2	20/20	2 weeks	a
Ye. (2012)	ME (0.5 mg/d) + DH (20 ml/d+250 mlNS)	14	67.3 ± 10.7	8/6	4 weeks	a
Ye. (2012)	ME (0.5 mg/d)	14	68.01 ± 10.99	7/7	4 weeks	a
ShaoXiong Cai (2013)	ME (2 mg/d+100 mlNS) + YXY (20 ml/d+250 mlNS)	100	—	54/46	4 weeks	a,b,c,d,e
ShaoXiong Cai (2013)	ME (2 mg/d+100 mlNS)	100	—	52/48	4 weeks	a,b,c,d,e
Jing Chen and Li. (2013)	ME (1 mg/d) + DH (30 ml/d)	36	43.4 ± 12.3	19/17	3 weeks	a
Jing Chen and Li. (2013)	ME (1 mg/d)	36	43.8 ± 11.9	16/20	3 weeks	a
XueMei Dai. (2013)	ME (1 mg/d) + YXY (25 ml/d+250 mlNS)	48	65.69 ± 12.37	28/20	2 weeks	a,b,c,d,e
XueMei Dai. (2013)	ME (1 mg/d)	48	64.72 ± 10.42	27/21	2 weeks	a,b,c,d,e
Feng. (2013)	ME (0.5 mg/d) + DH (30 ml/d+250 mlNS)	60	—	36/24	2 weeks	a,d,e
Feng. (2013)	ME (0.5 mg/d)	56	—	33/23	2 weeks	a,d,e
Genli Han and Wang. (2013)	ME (0.5 mg/d) + DH (20 ml/d+250 mlNS)	40	—	28/12	4 weeks	a
Genli Han and Wang. (2013)	ME (0.5 mg/d)	40	—	26/14	4 weeks	a
Hu. (2013)	ME (0.5 mg/d) + DH (20 ml/d+250 mlNS)	60	54.8 ± 8.2	36/24	2 weeks	a
Hu. (2013)	ME (0.5 mg/d)	60	53.1 ± 7.9	34/26	2 weeks	a
Hui Sun (2013)	ME (0.5 mg/d) + DSCXQ (10 ml/d+250 mlNS)	57	57.6 ± 7.8	30/27	3 weeks	a,b,c,d,e
Hui Sun (2013)	ME (0.5 mg/d)	57	59.1 ± 9.2	30/27	3 weeks	a,b,c,d,e
Zhou. (2013)	ME (0.5 mg/d) + YXY (25 ml/d+250 mlNS)	40	59 ± 11	24/16	2 weeks	a
Zhou. (2013)	ME (0.5 mg/d)	40	61 ± 12	22/18	2 weeks	a
An. (2014)	ME (0.5 mg/d) + DH (40 ml/d+250 mlNS)	53	36–68	29/24	2 weeks	a
An. (2014)	ME (0.5 mg/d)	53	31–67	31/22	2 weeks	a
Fang He et al. (2014)	ME (0.5 mg/d) + DH (40 ml/d+250 mlNS)	30	62.8 ± 3.7	19/11	4 weeks	b,c,d,e
Fang He et al. (2014)	ME (0.5 mg/d)	25	64.8 ± 3.9	14/11	4 weeks	b,c,d,e
Li. (2014)	ME (0.5 mg/d) + DH (20 ml/d+250 mlNS)	42	55.83 ± 13.07	24/18	4 weeks	a,b,c,d,e
Li. (2014)	ME (0.5 mg/d)	42	55.1 ± 13.94	20/22	4 weeks	a,b,c,d,e
Liao. (2014)	ME (1 mg/d) + DZHS(40 mg/d+250 mlNS)	48	59.3 ± 7.12	26/22	2 weeks	a,b,c,d,e
Liao. (2014)	ME (1 mg/d)	48	58.9 ± 6.38	28/20	2 weeks	a,b,c,d,e
WenLe Sui. (2014)	ME (0.5 mg/d+250 mlNS) + DH (20 ml/d+250 mlNS)	50	57.67 ± 8.89	27/23	4 weeks	a,b,c,d,e
WenLe Sui. (2014)	ME (0.5 mg/d+250 mlNS)	50	57.76 ± 8.98	24/26	4 weeks	a,b,c,d,e
Xia Wang and Zhu. (2014)	ME (0.5 mg/d+200 mlNS) + DH (20 ml/d+200 mlNS)	50	57.67 ± 8.89	25/25	4 weeks	a
Xia Wang and Zhu. (2014)	ME (0.5 mg/d+200 mlNS)	50	57.76 ± 8.98	25/25	4 weeks	a
Lei Xu and Chang. (2014)	ME (0.5 mg/d) + DH (20 ml/d+250 mlNS)	45	—	—	2 weeks	a
Lei Xu and Chang. (2014)	ME (0.5 mg/d)	45	—	—	2 weeks	a
Menghua Yang and Liu. (2022)	ME (1 mg/d) + GGS (20 mg/d+250 mlNS)	55	56 ± 8	29/26	4 weeks	a,b,c,d,e
Menghua Yang and Liu. (2022)	ME (1 mg/d)	55	56 ± 8	30/25	4 weeks	a,b,c,d,e
Li. (2015)	ME (0.5 mg/d) + DZHS(40 ml/d+250 mlNS)	30	—	—	2 weeks	a,c,e
Li. (2015)	ME (0.5 mg/d)	30	—	—	2 weeks	a,c,e
He Lu. (2015)	ME (0.5 mg/d) + YXY (20 ml/d+250 mlNS)	50	47.41 ± 6.25	27/23	4 weeks	d,e
He Lu. (2015)	ME (0.5 mg/d)	52	48.52 ± 6.52	28/24	4 weeks	d,e
WenJuanQiu nd Ding. (2015)	ME (2 mg/d+100 mlNS) + DH (40 ml/d+250 mlNS)	40	65 ± 2.3	17/13	2 weeks	a,b,c,d,e
WenJuanQiu nd Ding. (2015)	ME (2 mg/d+100 mlNS)	40	65 ± 2.4	20/20	2 weeks	a,b,c,d,e
Wang (2015a)	ME (0.5 mg/d+200 mlNS) + DH (20 ml/d+200 mlNS)	50	57.67 ± 8. 89	25/25	4 weeks	a
Wang (2015a)	ME (0.5 mg/d+200 mlNS)	50	57.76 ± 8. 98	25/25	4 weeks	a
Wang (2015a)	ME (0.5 mg/d) + DH (20 ml/d)	40	51.72 ± 7.38	23/17	2 weeks	a,b,c,d,e
Wang (2015a)	ME (0.5 mg/d)	40	52.11 ± 7.16	21/19	2 weeks	a,b,c,d,e
XiangXiang Zhou. (2015a)	ME (1 mg/d+250 mlNS) + DH (35 ml/d+250 mlNS)	62	67.5 ± 3.1	41/21	4 weeks	a,b,c,d,e
XiangXiang Zhou. (2015a)	ME (1 mg/d+250 mlNS)	62	68.2 ± 2.1	43/19	4 weeks	a,b,c,d,e
Jin Xie et al. (2016)	ME (2 mg/d+100 mlNS) + DH (40 ml/d+250 mlNS)	50	63 ± 1.9	27/23	4 weeks	a
Jin Xie et al. (2016)	ME (2 mg/d+100 mlNS)	50	62 ± 2.6	24/26	4 weeks	a
Yang et al. (2016)	ME (0.5 mg/d) + DH (30 ml/d+250 mlNS)	74	—	—	4 weeks	a
Yang et al. (2016)	ME (0.5 mg/d)	70	—	—	4 weeks	a
Zhang. (2016)	ME (0.5 mg/d+100 mlNS) + DSCXQ (15 ml/d+100 mS)	42	58.2 ± 6.8	26/16	2 weeks	a
Zhang. (2016)	ME (0.5 mg/d+100 mlNS)	42	56.3 ± 5.5	22/20	2 weeks	a
Hu. (2017)	ME (0.5 mg/d) + DH (20 ml/d+250 mlNS)	30	46.41 ± 6.45	16/14	4 weeks	a,d,e
Hu. (2017)	ME (0.5 mg/d)	30	47.52 ± 7.52	18/12	4 weeks	a,d,e
Li et al. (2017b)	ME (2 mg/d+100 mlNS) + YXY (20 ml/d+250 mlNS)	33	76.02 ± 4.18	20/13	4 weeks	a,b,c,d,e
Li et al. (2017b)	ME (2 mg/d+100 mlNS)	33	75.17 ± 4.09	19/14	4 weeks	a,b,c,d,e
Li. (2018)	ME (1 mg/d+250 mlNS) + DSCXQ (120 mg/d+250 mlNS)	43	51.3 ± 4.6	27/16	2 weeks	a,b,c,d,e
Li. (2018)	ME (1 mg/d+250 mlNS)	43	51.4 ± 4.7	26/17	2 weeks	a,b,c,d,e
Weiping Cao. (2019)	ME (0.5 mg/d) + YXY (20 ml/d+500 mlNS)	35	67.74 ± 4.35	18/17	4 weeks	a
Weiping Cao. (2019)	ME (0.5 mg/d)	35	67.57 ± 4.51	20/15	4 weeks	a
Zhao. (2019)	ME (0.5 mg/d) + YXY (25 ml/d+250 mlNS)	60	58. 3 ± 5.6	32/28	15 d	a,b,c,d,e
Zhao. (2019)	ME (0.5 mg/d)	60	57. 8 ± 5.8	31/29	15 d	a,b,c,d,e
Fang Wang. (2020)	ME (1 mg/d) + KDZ (40 ml/d+250 mlNS)	78	55.7 ± 3.2	45/33	4 weeks	a,b,c,d,e
Fang Wang. (2020)	ME (1 mg/d)	78	55.2 ± 3.5	44/34	4 weeks	a,b,c,d,e
Zhao et al. (2021)	ME (1.5 mg/d) + DSCXQ (15 ml/d)	30	51.29 ± 10.46	17/13	2 weeks	a
Zhao et al. (2021)	ME (1.5 mg/d)	30	50.73 ± 11.28	18/12	2 weeks	a

a: Overall response rate b: Median motor nerve conduction velocity c: median sensory nerve conduction velocity d: common peroneal motor nerve conduction velocity e: common peroneal sensory nerve conduction velocity.

ME, mecobalamin; DZXX, dengzhan xixin injection; KDZ, Kudiezi Injection; HH, Honghua Injection; DH, Danhong injection; YXY, Yinxingye injection; DSCXQ, Danshenchuanxiongqin injection; DZHS, Dengzhanhua injection; GGS, Gegensu injection.

### 3.3 Risk of bias of the included studies

**FIGURE 2:**
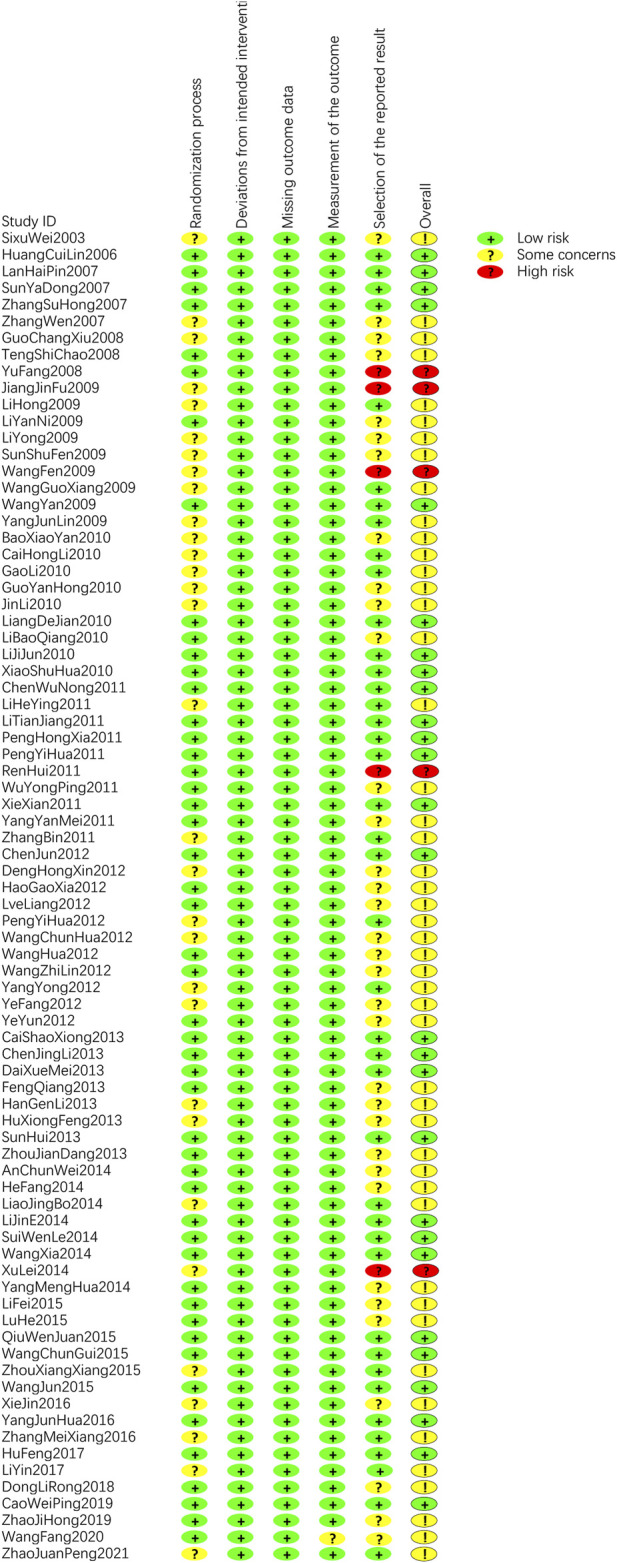
Risk of bias summary.

Although randomization was specified in all 80 articles, 28 articles (Si, 2003; Wen, 2007; Changxiu Guo and Sun, 2008; Hong Li and Liang, 2009; Shufen Sun, 2009; Wang et al., 2009; Yang, 2009; Yong Li, 2009; Bao, 2010; Gao and Zhang, 2010; Guo, 2010; Hongli Cai et al., 2010; Li Jin et al., 2010; Heying Li, 2011; Zhang, 2011; Chunhua Wang and Zhang, 2012b; Deng, 2012; Fangye and Liu, 2012; Peng et al., 2012; Yong Yang et al., 2012; Genli Han and Wang, 2013; Hu, 2013; Liao, 2014; Wang, 2015a; Jin Xie et al., 2016; Zhang, 2016; Ying Li, 2017; Zhao et al., 2021) did not clearly describe the methods of randomization, nor did they mention clear measures of concealment, which were rated as “some concerns”. There were 20 articles (Shichao Teng and Wang, 2008; Li, 2009; Li, 2010a; Wu, 2011; Yang, 2011; Liang Lv et al., 2012; Shao, 2012; Wang, 2012; Ye, 2012; Zhilin Wang, 2012; Feng, 2013; Zhou, 2013; An, 2014; Fang He et al., 2014; He Lu, 2015; Li, 2015; Lirong Dong et al., 2018; Zhao, 2019; Fang Wang, 2020; Menghua Yang and Liu, 2022) with incomplete reports on protocol analysis, which were rated as “some concerns”. There were 5 articles (Yu, 2008; Jiang, 2009; Fen Wang, 2009; Ren, 2011; Lei Xu and Chang, 2014) whose results were selectively reported and rated as “high risk”. In summary, the overall risk of 5 studies was “high risk” (Yu, 2008; Jiang, 2009; Fen Wang, 2009; Ren, 2011; Lei Xu and Chang, 2014), 48 studies was “some concerns”(Lirong Dong et al., 2018), (Genli Han and Wang, 2013), (Ye, 2012; Genli Han and Wang, 2013; Zhang, 2016; Zhao, 2019; Zhao et al., 2021), (Hong Li and Liang, 2009), (Li, 2010a; Bao, 2010; Gao and Zhang, 2010), (Yang, 2011), (Wang, 2012), (Fangye and Liu, 2012), (Zhou, 2013; He Lu, 2015; Ying Li, 2017), (Li, 2010b; Peng et al., 2012; Liao, 2014; Li, 2015), (Li, 2009; Shufen Sun, 2009; Wang et al., 2009; Yang, 2009; Yong Li, 2009; Guo, 2010; Hongli Cai et al., 2010), (Wu, 2011), (Chunhua Wang and Zhang, 2012b; Deng, 2012; Liang Lv et al., 2012; Shao, 2012; Yong Yang et al., 2012; Zhilin Wang, 2012), (Feng, 2013; Hu, 2013; An, 2014; Fang He et al., 2014), (Xiangxiang Zhou, 2015b), (Jin Xie et al., 2016), (Si, 2003), (Heying Li, 2011), (Menghua Yang and Liu, 2022), (Shichao Teng and Wang, 2008), (Wen, 2007; Changxiu Guo and Sun, 2008; Fang Wang, 2020), (Zhang, 2011) and the remaining studies were “low risk” (Huang, 2006; Haiping Lan, 2007; Sun et al., 2007; Zhang et al., 2007; Wang, 2009; Li, 2010b; Dejian Liang and Zhu, 2010; Xiao, 2010; Peng, 2011; Peng et al., 2011; Tianjiang Li, 2011; Wunong Chen and Li, 2011; XIan Xie et al., 2011; Chen, 2012; Hui Sun, 2013; Shaoxiong Cai et al., 2013; Jinli Chen and Li, 2013; Xuemei Dai, 2013; Li, 2014; Wenle Sui, 2014; Xia Wang and Zhu, 2014; Wang, 2015a; Wang, 2015b; Xiangxiang Zhou, 2015b; Wenjuan Qiu and Ding, 2015; Yang et al., 2016; Hu, 2017; Weiping Cao, 2019). The results of risk of bias for included studies are shown in Figure 2.

### 3.4 Network meta-analysis

**FIGURE 3:**
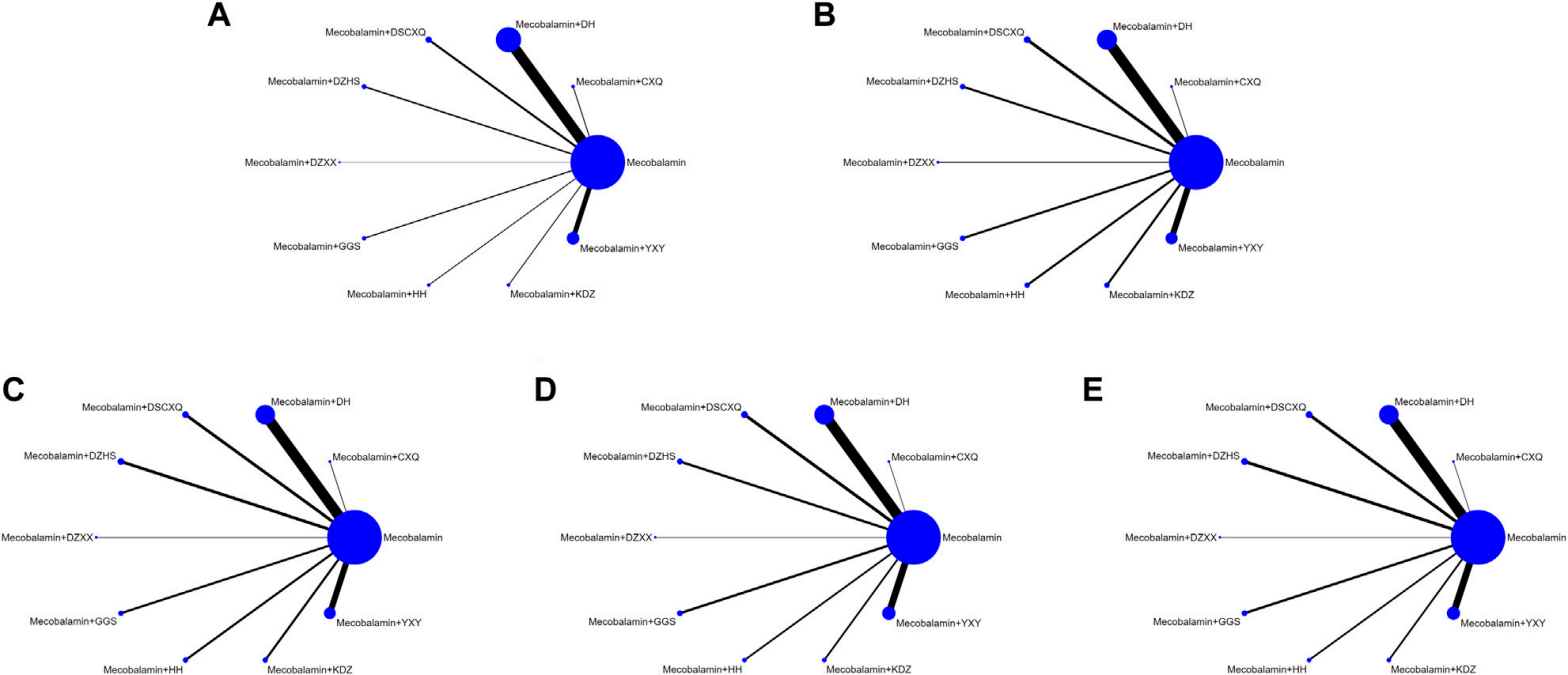
Network graphs of outcomes. **(A)** Overall response rate; **(B)** median motor nerve conduction velocity; **(C)** median sensory nerve conduction velocity; **(D)** common peroneal motor nerve conduction velocity; **(E)** common peroneal sensory nerve conduction velocity.

We performed a statistical analysis of all indicators using a random-effects model, with a total of 50,000 iterations, starting with the 20001st simulation. The network diagram is shown in Figure 3. The lines in the figure represent the interventions of direct comparison, the line thickness represents the number of studies, and the dot size represents the sample size of the intervention.

### 3.5 Primary indicators

#### 3.5.1 Overall response rate

A total of 76 studies reported the overall response rate. There were 4 studies on ME + GGS, 3 studies on ME + HH, 5 studies on ME + DZHS, 3 studies on ME + CXQ, 34 studies on ME + DH, 1 studies on ME + DZXX, 16 studies on ME + YXY, 7 studies on ME + DSCXQ, and 3 studies on ME + KDZ. Compared with mecobalamin alone, ME + CXQ [RR = 1.2, 95% CI (1.06,1.36)], ME + DH [RR = 1.29, 95% CI (1.23,1.35)], ME + DSCXQ [RR = 1.34, 95% CI (1.23,1.49)], ME + DZHS [RR = 1.29, 95% CI (1.15,1.46)], ME + DZXX [RR = 1.64, 95% CI (1.26,2.21)], ME + GGS [RR = 1.27, 95% CI (1.13,1.45)], ME + HH [RR = 1.43, 95% CI (1.2,1.74)], ME + KDZ [RR = 1.12. 95% CI (1,1.25)] and ME + YXY [RR = 1.3, 95% CI (1.22,1.39)] had better clinical efficacy (Table2).

**TABLE 2:** Network meta-analysis of overall response rate.

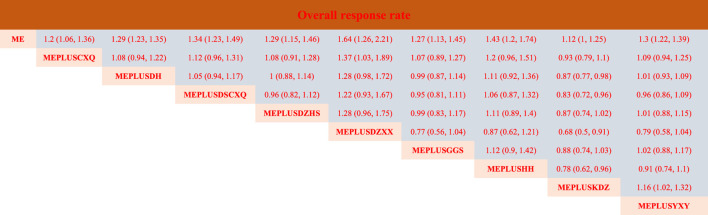
**ME**	1.2 (1.06, 1.36)	1.29 (1.23, 1.35)	1.34 (1.23, 1.49)	1.29 (1.15, 1.46)	1.64 (1.26, 2.21)	1.27 (1.13, 1.45)	1.43 (1.2, 1.74)	1.12 (1, 1.25)	1.3 (1.22, 1.39)
	**MEPLUSCXQ**	1.08 (0.94, 1.22)	1.12 (0.96, 1.31)	1.08 (0.91, 1.28)	1.37 (1.03, 1.89)	1.07 (0.89, 1.27)	1.2 (0.96, 1.51)	0.93 (0.79, 1.1)	1.09 (0.94, 1.25)
		**MEPLUSDH**	1.05 (0.94, 1.17)	1 (0.88, 1.14)	1.28 (0.98, 1.72)	0.99 (0.87, 1.14)	1.11 (0.92, 1.36)	0.87 (0.77, 0.98)	1.01 (0.93, 1.09)
			**MEPLUSDSCXQ**	0.96 (0.82, 1.12)	1.22 (0.93, 1.67)	0.95 (0.81, 1.11)	1.06 (0.87, 1.32)	0.83 (0.72, 0.96)	0.96 (0.86, 1.09)
				**MEPLUSDZHS**	1.28 (0.96, 1.75)	0.99 (0.83, 1.17)	1.11 (0.89, 1.4)	0.87 (0.74, 1.02)	1.01 (0.88, 1.15)
					**MEPLUSDZXX**	0.77 (0.56, 1.04)	0.87 (0.62, 1.21)	0.68 (0.5, 0.91)	0.79 (0.58, 1.04)
						**MEPLUSGGS**	1.12 (0.9, 1.42)	0.88 (0.74, 1.03)	1.02 (0.88, 1.17)
							**MEPLUSHH**	0.78 (0.62, 0.96)	0.91 (0.74, 1.1)
								**MEPLUSKDZ**	1.16 (1.02, 1.32)
									**MEPLUSYXY**

**FIGURE 4:**
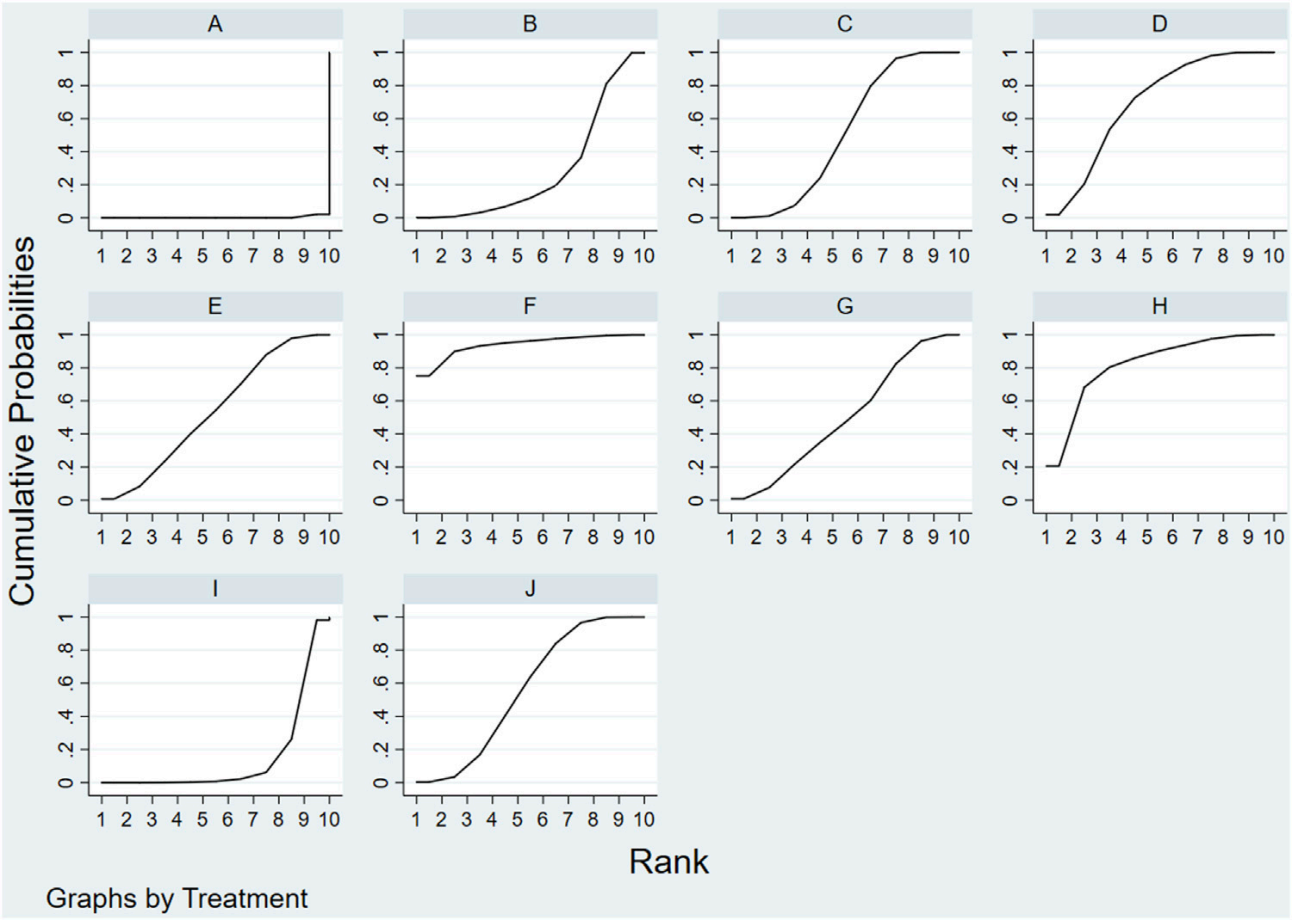
Rank of the cumulative probabilities for outcomes: overall response rate. **(A)**: ME; **(B)** ME + CXQ; **(C)** ME + DH; **(D)** ME + DSCXQ; **(E)** ME + DZHS; **(F)** ME + DZXX; **(G)** ME + GGS; **(H)** ME + HH; **(I)** ME + KDZ; **(J)** ME + YXY).

As shown in Figure 4 and Table 3, the ranking probabilities results of network-analysis in responder rate were as followed: ME + DZXX (93.9%) > ME + HH (81.8%) > ME + DSCXQ (69.2%) > ME + YXY (56.2%) > ME + DZHS(53.7%) > ME + DH (51.1%) > ME + GGS (50.1%) > ME + CXQ (28.8%) > ME + KDZ (14.9%).

**TABLE 3:** The cumulative ranking probabilities (SUCRA) results of overall response rate.

Intervention	Overall response rate	
	Sucar%	Rank
ME	0.2	10
ME + CXQ	28.8	8
ME + DH	51.1	6
ME + DSCXQ	69.2	3
ME + DZHS	53.7	5
ME + DZXX	93.9	1
ME + GGS	50.1	7
ME + HH	81.8	2
ME + KDZ	14.9	9
ME + YXY	56.2	4

### 3.6 Secondary indicators

#### 3.6.1 Median nerve conduction velocity

**FIGURE 5:**
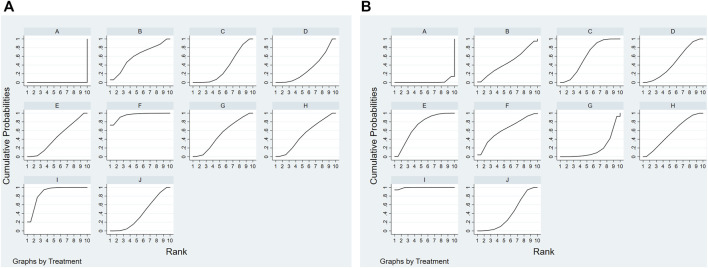
Rank of the cumulative probabilities for outcomes: Median nerve. **(A)** Median motor nerve conduction velocity; **(B)** median sensory nerve conduction velocity. [**(A)**: ME; **(B)** ME + CXQ; **(C)** ME + DH; **(D)** ME + DSCXQ; **(E)** ME + DZHS; **(F)** ME + DZXX; **(G)** ME + GGS; **(H)** ME + HH; **(I)** ME + KDZ; **(J)** ME + YXY].

Forty articles reported median motor nerve conduction velocity and 41 articles reported median sensory nerve conduction velocity. As for the median motor nerve conduction velocity, the ranking probabilities results (Table4) of network-analysis showed ME + DZXX (95.1%) > ME + KDZ (87.8%) > ME + CXQ (60.7%) > ME + GGS (51.6%) > ME + HH(50.9%) > ME + DZHS(45.6%) > ME + YXY (40.2%) > ME + DH (35.7%) > ME + DSCXQ (32.5%); with regards to the median sensory nerve conduction velocity, the ranking probabilities results were ME + KDZ (99.3%) > ME + DZHS(70.7%) > ME + DZXX (62.2%) > ME + DH (60.7%) > ME + HH(55.3%) > ME + CXQ (46.3%) > ME + DSCXQ (45.9%) > ME + YXY (38.9%) > ME + GGS (19%) (Figure 5 and Table 5).

**TABLE 4:** Network meta-analysis of median nerve.

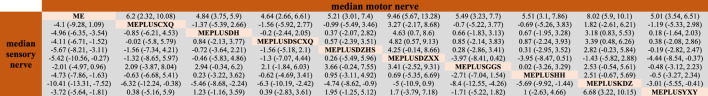
median sensory nerve	ME	6.2 (2.32, 10.08)	4.84 (3.75, 5.9)	4.64 (2.66, 6.61)	5.21 (3.01, 7.4)	9.46 (5.67, 13.28)	5.49 (3.23, 7.7)	5.51 (3.1, 7.86)	8.02 (5.9, 10.1)	5.01 (3.54, 6.51)
	−4.1 (−9.28, 1.09)	**MEPLUSCXQ**	−1.37 (−5.39, 2.66)	−1.56 (−5.92, 2.77)	−0.99 (−5.49, 3.46)	3.27 (−2.17, 8.68)	−0.7 (−5.22, 3.77)	−0.69 (−5.26, 3.83)	1.82 (−2.61, 6.21)	−1.19 (−5.33, 2.98)
	−4.96 (-6.35, −3.54)	−0.85 (−6.21, 4.53)	**MEPLUSDH**	−0.2 (−2.44, 2.05)	0.37 (−2.07, 2.82)	4.63 (0.7, 8.6)	0.66 (−1.83, 3.13)	0.67 (−1.95, 3.28)	3.18 (0.83, 5.53)	0.18 (−1.64, 2.03)
	−4.11 (-6.71, **-**1.52)	−0.02 (−5.8, 5.79)	0.84 (−2.13, 3.77)	**MEPLUSDSCXQ**	0.57 (−2.39, 3.51)	4.82 (0.57, 9.13)	0.85 (−2.14, 3.83)	0.87 (−2.24, 3.93)	3.39 (0.48, 6.26)	0.38 (−2.08, 2.86)
	−5.67 (−8.21, −3.11)	−1.56 (−7.34, 4.21)	−0.72 (−3.64, 2.21)	−1.56 (−5.18, 2.1)	**MEPLUSDZHS**	4.25 (−0.14, 8.66)	0.28 (−2.86, 3.41)	0.31 (−2.95, 3.52)	2.82 (−0.23, 5.84)	−0.19 (−2.82, 2.47)
	−5.42 (−10.56, −0.27)	−1.32 (−8.65, 5.97)	−0.46 (−5.83, 4.86)	−1.3 (−7.07, 4.44)	0.26 (−5.49, 5.96)	**MEPLUSDZXX**	−3.97 (−8.41, 0.42)	−3.95 (−8.47, 0.51)	−1.43 (−5.82, 2.88)	−4.44 (−8.54, -0.37)
	−2.01 (−4.97, 0.96)	2.09 (−3.87, 8.04)	2.94 (−0.34, 6.2)	2.1 (−1.84, 6.03)	3.66 (−0.24, 7.55)	3.41 (−2.52, 9.31)	**MEPLUSGGS**	0.02 (−3.26, 3.29)	2.53 (−0.54, 5.61)	−0.48 (−3.12, 2.23)
	−4.73 (−7.86, −1.63)	−0.63 (−6.68, 5.41)	0.23 (−3.22, 3.62)	−0.62 (−4.69, 3.41)	0.95 (−3.11, 4.92)	0.69 (−5.35, 6.69)	−2.71 (−7.04, 1.54)	**MEPLUSHH**	2.51 (−0.67, 5.69)	−0.5 (−3.27, 2.34)
	−10.41 (−13.31, −7.52)	−6.32 (−12.24, −0.38)	−5.46 (−8.68, −2.24)	−6.3 (−10.19, −2.42)	−4.74 (−8.62, −0.9)	−5 (−10.9, 0.9)	−8.4 (−12.55, −4.26)	−5.69 (−9.92, −1.44)	**MEPLUSKDZ**	−3.01 (−5.55, -0.41)
	−3.72 (−5.64, −1.81)	0.38 (−5.16, 5.9)	1.23 (−1.16, 3.59)	0.39 (−2.83, 3.61)	1.95 (−1.25, 5.12)	1.7 (−3.79, 7.18)	−1.71 (−5.22, 1.82)	1 (−2.63, 4.66)	6.68 (3.22, 10.15)	**MEPLUSYXY**

**TABLE 5:** The cumulative ranking probabilities (SUCRA) results of median nerve.

Intervention	Median motor nerve		Median sensory nerve	
	Sucar%	Rank	Sucar%	Rank
ME	0	10	1.6	10
ME + CXQ	60.7	3	46.3	6
ME + DH	35.7	8	60.7	4
ME + DSCXQ	32.5	9	45.9	7
ME + DZHS	45.6	6	70.7	2
ME + DZXX	95.1	1	62.2	3
ME + GGS	51.6	4	19	9
ME + HH	50.9	5	55.3	5
ME + KDZ	87.8	2	99.3	1
ME + YXY	40.2	7	38.9	8

#### 3.6.2 Common peroneal nerve conduction velocity

**FIGURE 6:**
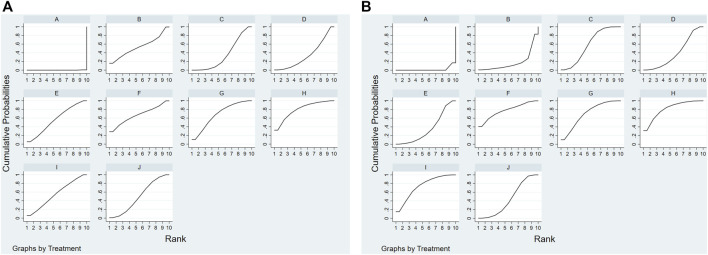
Rank of the cumulative probabilities for outcomes: Common peroneal nerve. **(A)** Common peroneal motor nerve conduction velocity; **(B)** common peroneal sensory nerve conduction velocity. [**(A)**: ME; **(B)** ME + CXQ; **(C)** ME + DH; **(D)** ME + DSCXQ; **(E)** ME + DZHS; **(F)** ME + DZXX; **(G)** ME + GGS; **(H)** ME + HH; **(I)** ME + KDZ; **(J)** ME + YXY].

There were 49 articles reporting common peroneal motor nerve conduction velocity and 50 articles reporting common peroneal sensory nerve conduction velocity (Table 6). The rankings results of common peroneal motor nerve conduction velocity were ME + HH(79.7%) > ME + GGS (68.4%) > ME + DZXX (67%) > ME + DZHS(56.8%) > ME + KDZ (55.3%) > ME + CXQ (53.9%) > ME + YXY (49.6%) > ME + DH (34.9%) > ME + DSCXQ (34.3%); meanwhile the rankings results of common peroneal sensory nerve conduction velocity were ME + HH(79.7%) > ME + DZXX (77.2%) > ME + KDZ (74.8%) > ME + GGS (70.4%) > ME + DH (58.3%) > ME + YXY (45%) > ME + DSCXQ (39.2%) > ME + DZHS(35.5%) > ME + CXQ (18.1%) (Figure 6 and Table 7).

**TABLE 6:** Network meta-analysis of common peroneal nerve.

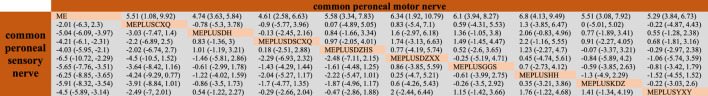
Common peroneal sensory nerve	ME	5.51 (1.08, 9.92)	4.74 (3.63, 5.84)	4.61 (2.58, 6.63)	5.58 (3.34, 7.83)	6.34 (1.92, 10.79)	6.1 (3.94, 8.27)	6.8 (4.13, 9.49)	5.51 (3.08, 7.92)	5.29 (3.84, 6.73)
	−2.01 (-6.3, 2.3)	MEPLUSCXQ	−0.78 (-5.3, 3.78)	−0.9 (−5.77, 3.96)	0.07 (−4.89, 5.05)	0.83 (−5.4, 7.1)	0.59 (−4.31, 5.53)	1.3 (−3.85, 6.47)	0 (−5.01, 5.02)	−0.22 (−4.87, 4.43)
	−5.04 (−6.09, -3.97)	−3.03 (−7.47, 1.4)	MEPLUSDH	−0.13 (−2.45, 2.16)	0.84 (−1.66, 3.34)	1.6 (−2.97, 6.18)	1.36 (−1.05, 3.8)	2.06 (−0.83, 4.96)	0.77 (−1.89, 3.41)	0.55 (−1.28, 2.38)
	−4.21 (−6.1, −2.31)	−2.2 (−6.89, 2.5)	0.83 (−1.36, 3)	MEPLUSDSCXQ	0.97 (−2.05, 4.01)	1.74 (−3.13, 6.63)	1.49 (−1.45, 4.47)	2.2 (−1.16, 5.55)	0.91 (−2.27, 4.05)	0.68 (−1.81, 3.16)
	−4.03 (−5.95, −2.1)	−2.02 (−6.74, 2.7)	1.01 (−1.19, 3.21)	0.18 (−2.51, 2.88)	MEPLUSDZHS	0.77 (−4.19, 5.74)	0.52 (−2.6, 3.65)	1.23 (−2.27, 4.7)	−0.07 (−3.37, 3.21)	−0.29 (−2.97, 2.38)
	−6.5 (−10.72, −2.29)	−4.5 (−10.5, 1.52)	−1.46 (-5.81, 2.86)	−2.29 (−6.93, 2.32)	−2.48 (−7.11, 2.15)	MEPLUSDZXX	−0.25 (−5.19, 4.71)	0.45 (−4.74, 5.61)	−0.84 (−5.89, 4.2)	−1.06 (−5.74, 3.59)
	−5.65 (−7.76, −3.51)	−3.64 (−8.42, 1.16)	−0.61 (−2.99, 1.78)	−1.43 (−4.29, 1.44)	−1.61 (−4.48, 1.25)	0.86 (−3.85, 5.59)	MEPLUSGGS	0.7 (−2.73, 4.12)	−0.59 (−3.85, 2.63)	−0.81 (−3.42, 1.79)
	−6.25 (−8.85, −3.65)	−4.24 (−9.29, 0.77)	−1.22 (−4.02, 1.59)	−2.04 (−5.27, 1.17)	−2.22 (−5.47, 1.01)	0.25 (−4.7, 5.21)	−0.61 (−3.99, 2.75)	MEPLUSHH	−1.3 (−4.9, 2.29)	−1.52 (−4.55, 1.52)
	−5.91 (−8.32, −3.54)	−3.91 (−8.84, 1.01)	−0.86 (−3.5, 1.73)	−1.7 (−4.77, 1.35)	−1.87 (−4.96, 1.17)	0.6 (−4.26, 5.43)	−0.26 (−3.5, 2.92)	0.35 (−3.21, 3.86)	MEPLUSKDZ	−0.22 (−3.03, 2.6)
	−4.5 (−5.89, −3.14)	−2.49 (−7, 2.01)	0.54 (−1.22, 2.27)	−0.29 (−2.66, 2.04)	−0.47 (−2.86, 1.88)	2 (−2.44, 6.44)	1.15 (−1.42, 3.66)	1.76 (−1.22, 4.68)	1.41 (−1.34, 4.19)	MEPLUSYXY

**TABLE 7:** The cumulative ranking probabilities (SUCRA) results of common peroneal nerve.

Intervention	Common peroneal motor nerve		Common peroneal sensory nerve	
	Sucar%	Rank	Sucar%	Rank
ME	0.1	10	1.9	10
ME + CXQ	53.9	6	18.1	9
ME + DH	34.9	8	58.3	5
ME + DSCXQ	34.3	9	39.2	7
ME + DZHS	56.8	4	35.5	8
ME + DZXX	67	3	77.2	2
ME + GGS	68.4	2	70.4	4
ME + HH	79.7	1	79.7	1
ME + KDZ	55.3	5	74.8	3
ME + YXY	49.6	7	45	6

#### 3.6.3 Consistency test

Since the network diagram had no closed loop, we performed a consistency test of the included articles. Except CXQ (*p* = 0.101 > 0.05), GGS (*p* = 0.16 > 0.05) in Median sensory nerve conduction velocity and CXQ (*p* = 0.34 > 0.05) in common peroneal sensory nerve conduction velocity, the *p* values for remaining indicators were all less than 0.05(*p* < 0.05), indicating significant inconsistency (Supplementary Materials S5–9).

#### 3.6.4 Safety

Adverse reactions were reported in 11 articles involving a total of 30 patients. Adverse reactions of YXY were reported in four articles, with a total of 11 patients (Li, 2010a; Bao, 2010; Shaoxiong Cai et al., 2013; Ying Li, 2017), the adverse events included 1 case of headache, 8 cases of dizziness, 2 cases of chest distress. Six studies reported adverse reactions of DH, with a total of 17 patients (Hongli Cai et al., 2010; Deng, 2012; Li, 2014; Wang, 2015a; Xiangxiang Zhou, 2015b; Wenjuan Qiu and Ding, 2015), the adverse events were 6 case of chest distress,5 case of dizziness, 2 cases of nausea, 1 case of xerostomia, 3 cases of flush. One article reported adverse reactions of HH(33), pruritus in 2 cases. Details of adverse reactions are shown in Table 8.

**TABLE 8:** Details of the adverse event occurred in the included studies.

Studies ID	Treatment	Intervention group	Control group
Si. (2003)	I:ME (0.5 mg/d+250 mlNS) + GGS (400 mg/d); C:ME (0.5 mg/d)	None	None
Huang. (2006)	I:ME (500 μg/d) + HH(20 ml + 500 mlNS); C:ME (500 μg/d)	None	None
HaiPing Lan. (2007)	I:ME (150 mg/d) + DZHS(150 mg/d+250 mlNS); C:ME (150 mg/d)	None	None
Sun et al. (2007)	I:ME (1500 μg/d) + CXQ (0.24 g/d); C:ME (1500 μg/d)	None	None
Sun et al. (2007)	I:ME (500 μg/d) + GGS (0.4 g/d+250 mlNS); C:ME (500 μg/d)	None	None
Zhang et al. (2007)	I:ME (1000 μg/d) + HH(40 mg/d); C:ME (1000 μg/d)	None	None
Guo. (2008)	I:ME (500 μg/d) + DH (20 ml/d+250 mlNS); C:ME (500 μg/d)	None	None
Shichao Teng and Wang. (2008)	I:ME (0.5 mg/d+100 ml) + KDZ (30 ml/d+250 mlNS); C:ME (0.5 mg/d+100 ml)	None	None
Yu. (2008)	I:ME (500 μg/d) + CXQ (200 mg/d+250 mlNS); C:ME (500 μg/d)	None	None
Jiang (2009)	I:ME (0.5 mg/d)+YXY (20 ml/d+250 mlNS); C:ME (0.5 mg/d)	None	None
Li. (2009)	I:ME (0.5 mg/d) + DZXX (20 ml/d+250 mlNS); C:ME (0.5 mg/d)	None	None
Li. (2009)	I:ME (0.5 mg/d) + DH (30 ml/d+250 mlNS); C:ME (0.5 mg/d)	None	None
Yong Li. (2009)	I:ME (1 mg/d)+DH (20 ml/d+250 mlNS); C:ME (1 mg/d)	None	None
ShuFen Sun. (2009)	I:ME (0.5 mg/d) + DH (20 ml/d+250 mlNS); C:ME (0.5 mg/d)	None	None
Wang. (2009)	I:ME (0.5 mg/d+250 mlNS) + YXY (20 ml/d+250 mlNS); C:ME (0.5 mg/d+250 mlNS)	None	None
Wang. (2009)	I:ME (0.5 mg/d) + DH (30 ml/d+250 mlNS); C:ME (0.5 mg/d)	None	None
Wang. (2009)	I:ME (0.5 mg/d+100 mlNS) + CXQ (80 ml/d+250 mlNS); C:ME (0.5 mg/d+100 mlNS)	None	None
Yang. (2009)	I:ME (0.5 mg/d) + DH (20 ml/d+200 mlNS); C:ME (0.5 mg/d)	None	None
Bao. (2010)	I:ME (0.5 mg/d+250 mlNS) + YXY (30 ml/d+250 mlNS); C:ME (0.5 mg/d+250 mlNS)	1 case of headache	None
Hongli Cai. (2010)	I:ME (2 mg/d+100 mlNS) + DH (40 ml/d+250 mlNS); C:ME (2 mg/d+100 mlNS)	1 case of chest distress, 1 case of dizziness	None
Gao and Zhang. (2010)	I:ME (0.5 mg/d) + YXY (20 ml/d+250 mlNS); C:ME (0.5 mg/d)	None	None
Guo. (2010)	I:ME (0.5 mg/d+150 mlNS) + DH (40 ml/d+250 mlNS); C:ME (0.5 mg/d+150 mlNS)	None	None
Li. (2010a)	I:ME (0.5 mg/d) + DZHS(20 ml/d+250 mlNS); C:ME (0.5 mg/d)	None	None
Li (2010b)	I:ME (0.5 mg/d+100 mlNS) + YXY (20 ml/d+250 mlNS); C:ME (0.5 mg/d+100 mlNS)	4 cases of dizziness	None
Li (2010b)	I:ME (0.5 mg/d) + DH (20 ml/d); C:ME (0.5 mg/d)	None	None
DeJian Liang and Zhu. (2010)	I:ME (0.5 mg/d) + HH(20 ml/d+500 mlNS); C:ME (0.5 mg/d)	2 cases of pruritus	None
Xiao. (2010)	I:ME (1.5 mg/d) + YXY (20 ml/d+250 mlNS); C:ME (1.5 mg/d)	None	None
Wunong Chen and Li. (2011)	I:ME (0.5 mg/d) + YXY (20 ml/d+250 mlNS); C:ME (0.5 mg/d)	None	None
Heying Li. (2011)	I:ME (0.5 mg/d)+GGS (300 ml/d+250 mlNS); C:ME (0.5 mg/d)	None	None
TianJiang Li. (2011)	I:ME (0.5 mg/d) + DSCXQ (10 ml/d+250 mlNS); C:ME (0.5 mg/d)	None	None
Peng. (2011)	I:ME (0.5 mg/d) + DSCXQ (20 ml/d+250 mlNS); C:ME (0.5 mg/d)	None	None
Peng et al. (2011)	I:ME (1 mg/d) + KDZ (30 ml/d+100 mlNS); C:ME (1 mg/d)	None	None
Ren. (2011)	I:ME (0.5 mg/d+100 mlNS) + DH (30 ml/d+250 mlNS); C:ME (0.5 mg/d+100 mlNS)	None	None
Wu. (2011)	I:ME (0.5 mg/d) + DH (20 ml/d+250 mlNS); C:ME (0.5 mg/d)	None	None
Xian Xie et al. (2011)	I:ME (0.5 mg/d+100 mlNS) + DSCXQ (15 ml/d+250 mlNS); C:ME (0.5 mg/d+100 mlNS)	None	None
Yang. (2011)	I:ME (0.5 mg/d+250 mlNS) + YXY (20 ml/d+250 mlNS); C:ME (0.5 mg/d+250 mlNS)	None	None
Zhang and Yue. (2011)	I:ME (1 mg/d+100 mlNS) + DH (30 ml/d+250 mlNS); C:ME (1 mg/d+100 mlNS)	None	None
Chen. (2012)	I:ME (0.5 mg/d) + YXY (20 ml/d+250 mlNS); C:ME (0.5 mg/d)	None	None
Deng. (2012)	I:ME (0.5 mg/d) + DH (20 ml/d+250 mlNS)C:ME (0.5 mg/d)	2 cases of nausea, 1 case of xerostomia	1 case of nausea, 1 case of xerostomia, 1 case of skin rash
Liang Lv et al. (2012)	I:ME (1 mg/d+20 mlNS) + DH (40 ml/d+100 mlNS); C:ME (1 mg/d+20 mlNS)	None	None
Peng et al. (2012)	I:ME (1 mg/d) + DZHS(75 mg/d+100 mlNS); C:ME (1 mg/d)	None	None
Shao. (2012)	I:ME (0.5 mg/d)+DH (20 ml/d+250 mlNS); C:ME (0.5 mg/d)	None	None
ChunHua Wang and Zhang. (2012b)	I:ME (1 mg/d+250 mlNS) + DH (20 ml/d+250 mlNS); C:ME (1 mg/d+250 mlNS)	None	None
Wang. (2012)	I:ME (0.5 mg/d) + YXY (30 ml/d+250 mlNS); C:ME (0.5 mg/d)	None	None
Zhilin Wang. (2012)	I:ME (1 mg/d) + DH(20 ml/d+250 mlNS); C:ME (1 mg/d)	None	None
Yong Yang et al. (2012)	I:ME (0.5 mg/d) + DH (20 ml/d+250 mlNS); C:ME (0.5 mg/d)	None	None
Ye. (2012)	I:ME (0.5 mg/d) + YXY (20 ml/d+250 mlNS); C:ME (0.5 mg/d)	None	None
Ye. (2012)	I:ME (0.5 mg/d) + DH (20 ml/d+250 mlNS); C:ME (0.5 mg/d)	None	None
ShaoXiong Cai (2013)	I:ME (2 mg/d+100 mlNS) + YXY (20 ml/d+250 mlNS); C:ME (2 mg/d+100 mlNS)	2 cases of chest distress, 2 cases of dizziness	None
Jing Chen and Li. (2013)	I:ME (1 mg/d) + DH (30 ml/d); C:ME (1 mg/d)	None	None
XueMei Dai. (2013)	I:ME (1 mg/d)+YXY (25 ml/d+250 mlNS); C:ME (1 mg/d)	None	None
Feng. (2013)	I:ME (0.5 mg/d) + DH (30 ml/d+250 mlNS); C:ME (0.5 mg/d)	None	None
Genli Han and Wang. (2013)	I:ME (0.5 mg/d) + DH (20 ml/d+250 mlNS); C:ME (0.5 mg/d)	None	None
Hu. (2013)	I:ME (0.5 mg/d) + DH (20 ml/d+250 mlNS); C:ME (0.5 mg/d)	None	None
Hui Sun (2013)	I:ME (0.5 mg/d) + DSCXQ (10 ml/d+250 mlNS); C:ME (0.5 mg/d)	None	None
Zhou. (2013)	I:ME (0.5 mg/d) + YXY (25 ml/d+250 mlNS); C:ME (0.5 mg/d)	None	None
An. (2014)	I:ME (0.5 mg/d) + DH (40 ml/d+250 mlNS); C:ME (0.5 mg/d)	None	None
Fang He et al. (2014)	I:ME (0.5 mg/d) + DH (40 ml/d+250 mlNS); C:ME (0.5 mg/d)	None	None
Li. (2014)	I:ME (0.5 mg/d) + DH (20 ml/d+250 mlNS); C:ME (0.5 mg/d)	3 cases of flush	1 case of diarrhea
Liao. (2014)	I:ME (1 mg/d) + DZHS(40 mg/d+250 mlNS); C:ME (1 mg/d)	None	None
WenLe Sui. (2014)	I:ME (0.5 mg/d+250 mlNS) + DH (20 ml/d+250 mlNS); C:ME (0.5 mg/d+250 mlNS)	None	None
Xia Wang and Zhu. (2014)	I:ME (0.5 mg/d+200 mlNS) + DH (20 ml/d+200 mlNS); C:ME (0.5 mg/d+200 mlNS)	None	None
Lei Xu and Chang. (2014)	I:ME (0.5 mg/d) + DH (20 ml/d+250 mlNS); C:ME (0.5 mg/d)	None	None
Menghua Yang and Liu. (2022)	I:ME (1 mg/d) + GGS (20 mg/d+250 mlNS); C:ME (1 mg/d)	None	None
Li. (2015)	I:ME (0.5 mg/d) + DZHS(40 ml/d+250 mlNS); C:ME (0.5 mg/d)	None	None
He Lu. (2015)	I:ME (0.5 mg/d) + YXY (20 ml/d+250 mlNS); C:ME (0.5 mg/d)	None	None
WenJuanQiu nd Ding (2015)	I:ME (2 mg/d+100 mlNS) + DH (40 ml/d+250 mlNS); C:ME (2 mg/d+100 mlNS)	2 cases of chest distress, 1 case of dizziness	2 cases of chest distress, 3 cases of dizziness
Wang (2015a)	I:ME (0.5 mg/d+200 mlNS) + DH (20 ml/d+200 mlNS); C:ME (0.5 mg/d+200 mlNS)	None	None
Wang (2015a)	I:ME (0.5 mg/d) + DH (20 ml/d); C:ME (0.5 mg/d)	1 case of chest distress, 2 cases of dizziness	1 case of chest distress
XiangXiang Zhou. (2015a)	I:ME (1 mg/d+250 mlNS) + DH (35 ml/d+250 mlNS); C:ME (1 mg/d+250 mlNS)	2 cases of chest distress, 1 case of dizziness	1 case of chest distress, 1 case of dizziness
Jin Xie et al. (2016)	I:ME (2 mg/d+100 mlNS) + DH (40 ml/d+250 mlNS); C:ME (2 mg/d+100 mlNS)	None	None
Yang et al. (2016)	I:ME (0.5 mg/d)+DH (30 ml/d+250 mlNS); C:ME (0.5 mg/d)	None	None
Zhang. (2016)	I:ME (0.5 mg/d+100 mlNS) + DSCXQ (15 ml/d+100 mlNS); C:ME (0.5 mg/d+100 mlNS)	None	None
Hu. (2017)	I:ME (0.5 mg/d) + DH (20 ml/d+250 mlNS); C:ME (0.5 mg/d)	None	None
Li et al. (2017b)	I:ME (2 mg/d+100 mlNS) + YXY (20 ml/d+250 mlNS); C:ME (2 mg/d+100 mlNS)	2 cases of dizziness	None
Li. (2018)	I:ME (1 mg/d+250 mlNS) + DSCXQ (120 mg/d+250 mlNS); C:ME (1 mg/d+250 mlNS)	None	None
Weiping Cao. (2019)	I:ME (0.5 mg/d) + YXY (20 ml/d+500 mlNS); C:ME (0.5 mg/d)	None	None
Zhao. (2019)	I:ME (0.5 mg/d) + YXY (25 ml/d+250 mlNS); C:ME (0.5 mg/d)	None	None
Fang Wang. (2020)	I:ME (1 mg/d) + KDZ (40 ml/d+250 mlNS); C:ME (1 mg/d)	None	None
Zhao et al. (2021)	I:ME (1.5 mg/d) + DSCXQ (15 ml/d); C:ME (1.5 mg/d)	None	None

#### 3.6.5 Publication bias

**FIGURE 7:**
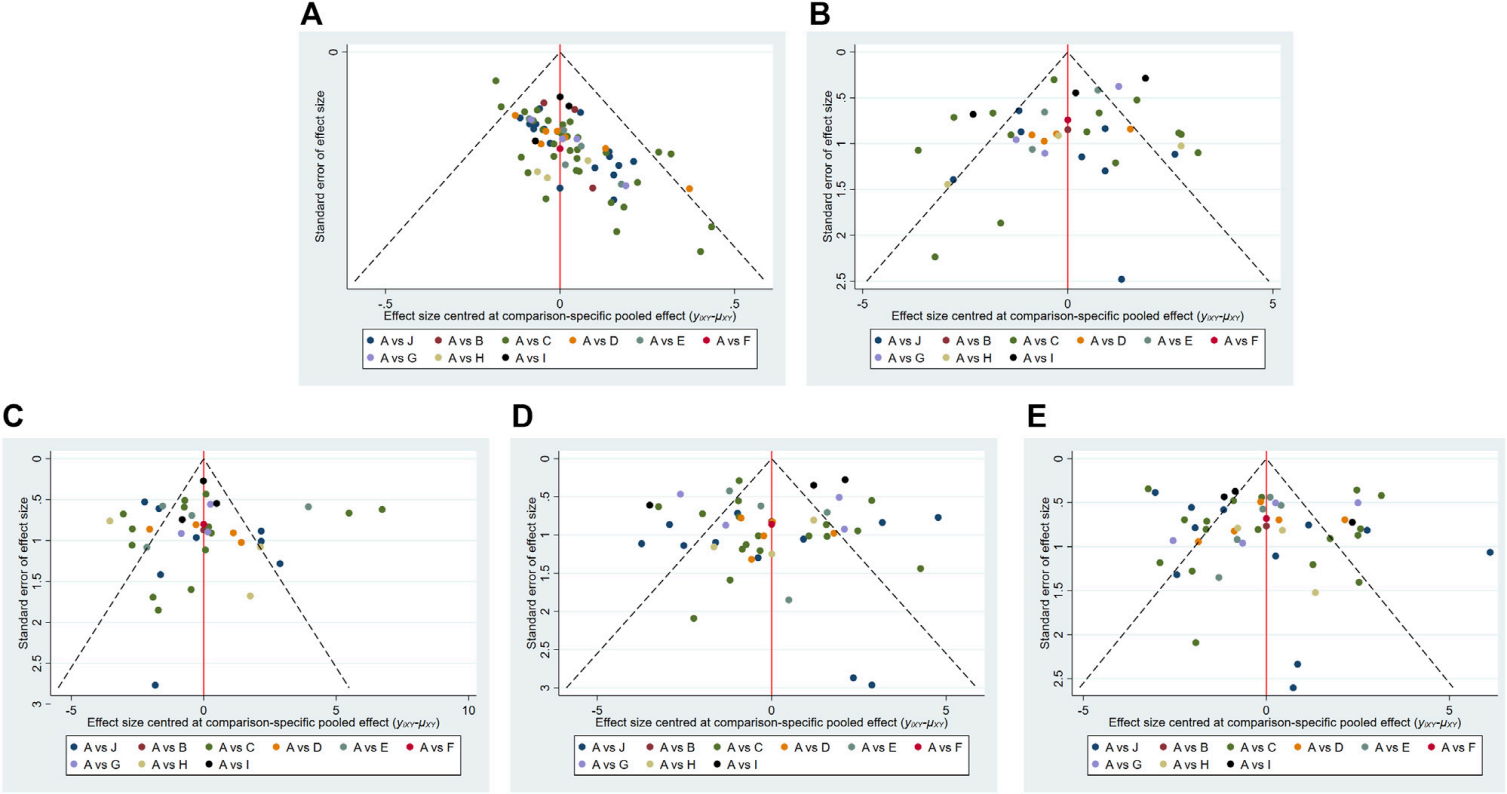
Funnel plots of outcomes. **(A)** Overall response rate; **(B)** median motor nerve conduction velocity; **(C)** median sensory nerve conduction velocity; **(D)** common peroneal motor nerve conduction velocity; **(E)** common peroneal sensory nerve conduction velocity. [**(A)**: ME; **(B)** ME + CXQ; **(C)** ME + DH; **(D)** ME + DSCXQ; **(E)** ME + DZHS; **(F)** ME + DZXX; **(G)** ME + GGS; **(H)** ME + HH; **(I)** ME + KDZ; **(J)** ME + YXY].

The funnel plots of overall response rate, median motor nerve conduction velocity, median sensory nerve conduction velocity, common peroneal motor nerve conduction velocity and common peroneal sensory nerve conduction velocity were visually asymmetric, which indicated that publication bias existed in these outcomes (Figure 7).

### 3.7 Sensitivity analysis

Sensitivity analysis was conducted by excluding each trial individually, and the results indicated that the findings were robust.

## 4 Discussion

The present systematic review and network meta-analysis included 80 studies, the overall risk of bias of 27 were consider as “low risk”, 48 studies were “some concerns” and 5 studies were “high risk”. The results of the network meta-analysis indicated that ME + DZXX was ranking first in improving overall response rate and the median motor nerve conduction velocity. ME + KDZ was ranking first in increasing the conduction velocity of median sensory nerve. ME + HH was ranking first in enhancing the conduction velocity of common peroneal motor nerve and common peroneal sensory nerve.

According to the Results, ME + DZXX was ranking first in terms of overall response rate and median motor nerve conduction velocity. DZXX was approved by the State Drug Administration in 2001. Pharmacological studies indicated that breviscapin in DZXX could reduce platelet damage, improve *in vivo* activity and exert neuroprotective effect (Li et al., 2017a; Shi et al., 2020). The scutellarin in DZXX have anti-inflammatory effects by blocking the expression of inflammatory genes (e.g., TNF-α, IL-6, NF-κB) (Lin et al., 2019) and inhibiting the TLR4/NF-κB signaling pathway (Zhang et al., 2017). Haiting A et al. found that the effect of DZXX on DPN patients was associated with the protective effect of neuronal mitochondria (An et al., 2021). Meanwhile, Xi J et al. discovered that scutellarin in DZXX could protect vascular endothelial cells from hyperglycemic injury by upregulating mitochondrial autophagy *via* the PINK1/Parkin signaling pathway (Xi et al., 2021). Among included RCTs, no adverse reactions of DZXX were reported. However, relevant studies suggested that some allergic reactions occured in DZXX (Xin et al., 2011; Li and Xie, 2012), which required to be confirmed in future.

The results revealed that ME + HH was the first in increasing common peroneal motor nerve conduction velocity and common peroneal sensory nerve conduction velocity and ME + KDZ was the first in improving median sensory nerve conduction velocity. Several studies concluded that KDZ and HH could promoted blood circulation and improved the circulatory system (Wang et al., 2018; Yu et al., 2018; Lu et al., 2021). In TCM theory, safflower has the effect of invigorating blood circulation, removing blood stasis and relieving pain, which is also in line with the treatment principle of “arthralgia syndrome” in TCM (Zhang, 2010). Li et al. (2017b) reported safflower extract could reduce neurological damage caused by DPN and its protective effect might be related to the promotion of VEGF-B, NGF, and GDNF expression. Consistent with our study, a meta-analysis revealed that the Kudiezi Injection was beneficial for DPN. However, more high-quality trials were needed to verify its efficacy and safety. (Li, 2018).

## 5 Limitations

The present systematic review and network meta-analysis had some limitations. First, RCTs of relevant Chinese medicine injections rarely reported adverse reactions, thus it is difficult to confirm the safety of Chinese medicine injections. Second, only one article focused on DZXX and three articles on HH were included. Due to limited RCTs, further clinical trials on DZXX and HH are needed to support our findings. Third, most of included RCTs had some concerns of bias, particularly in the randomization and selection of the reported result. The clinical trials should pay attention to controlling risk of bias.

## 6 Conclusion

This study determined the efficacy of nine Chinese medicine injections combined with mecobalamin. DZXX may be the best adjunctive Chinese medicine injection for DPN patients. Due to potential risk of bias and limited RCTs, the results need to be treated with cautions.

## Data Availability

The original contributions presented in the study are included in the article/Supplementary Material, further inquiries can be directed to the corresponding author.
