# Supplementary material for: The efficacy and safety of mecobalamin combined with Chinese medicine injections in the treatment of diabetic peripheral neuropathy: A systematic review and Bayesian network meta-analysis of randomized controlled trials
Source: Front Pharmacol. 2022 Nov 4;13:957483. doi: 10.3389/fphar.2022.957483 (PMC9672474; doi:10.3389/fphar.2022.957483)
Supplement: Supplementary file 1 [file DataSheet2.PDF]

Injection	Originates from	Family	Drug form	
Dengzhan xixin injection	Erigeron breviscapus (the whole herbal)	Asteraceae	dried herb	
Kudiezi Injection	Crepidiastrum denticulatum	Asteraceae	dried herb	
Safflower injection	Carthamus tinctorius	Asteraceae	dried flower	
Puerarin injection	Pueraria montana	Fabaceae	dried root, dried flowers and buds	
Ginko bilboa leaves injection	Ginkgo biloba	Ginkgoaceae	whole or fragmented, dried leaf	
Breviscapin injection	Erigeron breviscapus (a extract of further separation)	Asteraceae	dried herb	
Ligustrazine injection	Ligusticum wallichii	Apiaceae	rhizome	
Salvia miltiorrhiza and Ligustrazine injection	Salvia miltiorrhiza	Lamiaceae	dried root and rhizome	*Salvia miltiorrhiza and Ligustrazine injection originates from Salvia miltiorrhiza and ligustrazine*
	Ligusticum striatum	Apiaceae	rhizome	
Danhong injection	Salvia miltiorrhiza	Lamiaceae	dried root and rhizome	*Danhong injection originates from Salvia miltiorrhiza and Carthamus tinctorius*
	Carthamus tinctorius	Asteraceae	dried flower	
