# Supplementary material for: The efficacy and safety of mecobalamin combined with Chinese medicine injections in the treatment of diabetic peripheral neuropathy: A systematic review and Bayesian network meta-analysis of randomized controlled trials
Source: Front Pharmacol. 2022 Nov 4;13:957483. doi: 10.3389/fphar.2022.957483 (PMC9672474; doi:10.3389/fphar.2022.957483)
Supplement: Supplementary file 2 [file DataSheet4.DOCX]

**Supplementary material 4:** Detailed information on Chinese medicine injections.

| **Injection** | **Scientific name of the plant** | **Species** | **Drug form** | **Composition with Chinese pinyin** |
| --- | --- | --- | --- | --- |
| Dengzhan xixin injection(DZXX) | *Erigeron breviscapus*(Vaniot) Hand.-Mazz[Asteraceae] | Erigerontis herba | Dried botanical drug | Dengzhanxixin |
| Chuanxiongqin injection(CXQ) | *Conioselinum anthriscoides 'Chuanxiong'*[Apiaceae] | Chuanxiong rhizoma | Dried rhizome | chuanxiongqin |
| Kudiezi injection(KDZ) | *Thlaspi arvense* L[Brassicaceae] | Thlaspi herba | Dried aerial part | Kudiezi |
| Honghua injection(HH) | *Carthamus tinctorius* L[Asteraceae] | Carthami flos | Dried flower | Honghua |
| Yinxingye injection(YXY) | *Ginkgo biloba* L[Ginkgoaceae] | Ginkgo folium | Dried leaf | Yinxingye |
| Gegensu injection(GGS) | *Pueraria montana var. thomsonii*(Benth.) M.R.Almeida[Fabaceae] | Puerariae thomsonii radix | Dried root | Gegensu |
| Dengzhanhua injection(DZHS) | *Erigeron breviscapus*(Vaniot) Hand.-Mazz[Asteraceae] | Erigerontis herba | Dried botanical drug | Dengzhanhua |
| Danshenchuanxiongqin injection(DSCXQ) | *Salvia miltiorrhiza* Bunge[Lamiaceae] | Salviae miltiorrhizae radix et rhizoma | Dried root and rhizome | Danshen, Chuanxiongqin |
|  | *Conioselinum anthriscoides 'Chuanxiong'*[Apiaceae] | Chuanxiong rhizoma | Dried rhizome |  |
| Danhong injection(DH) | *Salvia miltiorrhiza* Bunge[Lamiaceae] | Salviae miltiorrhizae radix et rhizoma | Dried root and rhizome | Danshen, Honghua |
|  | *Carthamus tinctorius* L[Asteraceae] | Carthami flos | Dried flower |  |
