# Supplementary material for: The efficacy and safety of mecobalamin combined with Chinese medicine injections in the treatment of diabetic peripheral neuropathy: A systematic review and Bayesian network meta-analysis of randomized controlled trials
Source: Front Pharmacol. 2022 Nov 4;13:957483. doi: 10.3389/fphar.2022.957483 (PMC9672474; doi:10.3389/fphar.2022.957483)
Supplement: Supplementary file 3 [file DataSheet7.DOCX]

**SUPPLEMENTARY MATERIAL 10:** Consistency test of common peroneal sensory nerve.

| **Intervention** | **P** | **SD** | **MD(95%CI)** |
| --- | --- | --- | --- |
| ME+CXQ VS ME | 0.101 | 2.3451844 | 4.1(-0.804, 9.004) |
| ME+DH VS ME | ＜0.0001 | 2.3451844 | 4.96(3.62, 6.29) |
| ME+DSCXQ VS ME | 0.001 | 2.3451844 | 4.11(1.64, 6.57) |
| ME+DZHS VS ME | ＜0.0001 | 2.3451844 | 5.66(3.26, 8.08) |
| ME+DZXX VS ME | 0.029 | 2.3451844 | 5.42(0.561, 10.27) |
| ME+GGS VS ME | 0.16 | 2.3451844 | 2.01(-0.79, 4.81) |
| ME+HH VS ME | 0.002 | 2.3451844 | 4.72(1.75, 7.701) |
| ME+KDZ VS ME | ＜0.0001 | 2.3451844 | 10.41(7.685, 13.13) |
| ME+YXY VS ME | ＜0.0001 | 2.3451844 | 3.73(1.917, 5.556) |
