# Supplementary material for: The efficacy and safety of mecobalamin combined with Chinese medicine injections in the treatment of diabetic peripheral neuropathy: A systematic review and Bayesian network meta-analysis of randomized controlled trials
Source: Front Pharmacol. 2022 Nov 4;13:957483. doi: 10.3389/fphar.2022.957483 (PMC9672474; doi:10.3389/fphar.2022.957483)
Supplement: Supplementary file 4 [file DataSheet6.DOCX]

**Supplementary material 9:** Consistency test of median motor nerve conduction velocity.

| **Intervention** | **P** | **SD** | **MD(95%CI)** |
| --- | --- | --- | --- |
| ME+CXQ VS ME | 0.001 | 1.6642479 | 6.20(2.54, 9.85) |
| ME+DH VS ME | ＜0.0001 | 1.6642479 | 4.83(3.82, 5.85) |
| ME+DSCXQ VS ME | ＜0.0001 | 1.6642479 | 4.64(2.79, 6.50) |
| ME+DZHS VS ME | ＜0.0001 | 1.6642479 | 5.20(3.14, 7.26) |
| ME+DZXX VS ME | ＜0.0001 | 1.6642479 | 9.46(5.89, 13.03) |
| ME+GGS VS ME | ＜0.0001 | 1.6642479 | 5.49(3.39, 7.60) |
| ME+HH VS ME | ＜0.0001 | 1.6642479 | 5.50(3.23, 7.78) |
| ME+KDZ VS ME | ＜0.0001 | 1.6642479 | 8.02(6.05, 9.98) |
| ME+YXY VS ME | ＜0.0001 | 1.6642479 | 5.02(3.60, 6.43) |
