# Supplementary material for: The efficacy and safety of mecobalamin combined with Chinese medicine injections in the treatment of diabetic peripheral neuropathy: A systematic review and Bayesian network meta-analysis of randomized controlled trials
Source: Front Pharmacol. 2022 Nov 4;13:957483. doi: 10.3389/fphar.2022.957483 (PMC9672474; doi:10.3389/fphar.2022.957483)
Supplement: Supplementary file 5 [file DataSheet3.DOCX]

**Supplementary material 3:** Injections' details of the included articles.

| **Studies** | **Injection** | **Source** | **Species** | **Main components** | **Dose** | **Quality control reported? (Y/N)** | **Chemial analysis reported? (Y/ N)** |
| --- | --- | --- | --- | --- | --- | --- | --- |
| Li et al.(2009) | Dengzhan xixin injection(DZXX) | YUNNAN BIOVALLEY PHARMACEUTICAL CO.,LTD | Erigerontis herba | Caffeic acid esters; Scutellarin | 10ml/Ampullen 10Ampullen/box | Y-Prepared according to Chinese pharmacopeia(2010) | Y-HPLC |
| Li et al.(2010) | Danhong injection(DH) | Not mentioned | Salviae miltiorrhizae radix et rhizoma; Carthami flos | / | / | / | / |
| Wang et al.(2009) | Danhong injection(DH) | Not mentioned | Salviae miltiorrhizae radix et rhizoma; Carthami flos | / | / | / | / |
| Wang et al.(2015) | Danhong injection(DH) | SHANDONG BUCHANG PHARMACEUTICALS CO., LTD | Salviae miltiorrhizae radix et rhizoma; Carthami flos | Sodium danshensu; Protocatechuic aldehyde; 4-Hydroxycinnamic acid; Rosmarinic acid; Salvianolic acid B | 10ml/Ampullen;  6Ampullen/box | Y-Prepared according to Chinese pharmacopeia(2010) | Y-HPLC |
| Wang et al.(2014) | Danhong injection(DH) | Not mentioned | Salviae miltiorrhizae radix et rhizoma; Carthami flos | / | / | / | / |
| Sun et al.(2009) | Danhong injection(DH) | Not mentioned | Salviae miltiorrhizae radix et rhizoma; Carthami flos | / | / | / | / |
| Li et al.(2014) | Danhong injection(DH) | Not mentioned | Salviae miltiorrhizae radix et rhizoma; Carthami flos | / | / | / | / |
| Han et al.(2013) | Danhong injection(DH) | Not mentioned | Salviae miltiorrhizae radix et rhizoma; Carthami flos | / | / | / | / |
| Qiu et al.(2015) | Danhong injection(DH) | SHANDONG BUCHANG PHARMACEUTICALS CO., LTD | Salviae miltiorrhizae radix et rhizoma; Carthami flos | Sodium danshensu; Protocatechuic aldehyde; 4-Hydroxycinnamic acid; Rosmarinic acid; Salvianolic acid B | 10ml/Ampullen;  6Ampullen/box | Y-Prepared according to Chinese pharmacopeia(2010) | Y-HPLC |
| Zhou et al.(2015) | Danhong injection(DH) | SHANDONG BUCHANG PHARMACEUTICALS CO., LTD | Salviae miltiorrhizae radix et rhizoma; Carthami flos | Sodium danshensu; Protocatechuic aldehyde; 4-Hydroxycinnamic acid; Rosmarinic acid; Salvianolic acid B | 10ml/Ampullen;  6Ampullen/box | Y-Prepared according to Chinese pharmacopeia(2010) | Y-HPLC |
| Hu et al.(2017) | Danhong injection(DH) | Not mentioned | Salviae miltiorrhizae radix et rhizoma; Carthami flos | / | / | / | / |
| Yang et al.(2009) | Danhong injection(DH) | SHANDONG BUCHANG PHARMACEUTICALS CO., LTD | Salviae miltiorrhizae radix et rhizoma; Carthami flos | Sodium danshensu; Protocatechuic aldehyde; 4-Hydroxycinnamic acid; Rosmarinic acid; Salvianolic acid B | 10ml/Ampullen;  6Ampullen/box | Y-Prepared according to Chinese pharmacopeia(2010) | Y-HPLC |
| Shao et al.(2012) | Danhong injection(DH) | Not mentioned | Salviae miltiorrhizae radix et rhizoma; Carthami flos | / | / | / | / |
| Zhang et al.(2011) | Danhong injection(DH) | Not mentioned | Salviae miltiorrhizae radix et rhizoma; Carthami flos | / | / | / | / |
| Wu et al.(2011) | Danhong injection(DH) | Not mentioned | Salviae miltiorrhizae radix et rhizoma; Carthami flos | / | / | / | / |
| Wang et al.(2012) | Danhong injection(DH) | SHANDONG BUCHANG PHARMACEUTICALS CO., LTD | Salviae miltiorrhizae radix et rhizoma; Carthami flos | Sodium danshensu; Protocatechuic aldehyde; 4-Hydroxycinnamic acid; Rosmarinic acid; Salvianolic acid B | 10ml/Ampullen;  6Ampullen/box | Y-Prepared according to Chinese pharmacopeia(2010) | Y-HPLC |
| Xu et al.(2014) | Danhong injection(DH) | Not mentioned | Salviae miltiorrhizae radix et rhizoma; Carthami flos | / | / | / | / |
| Li et al.(2009) | Danhong injection(DH) | SHANDONG BUCHANG PHARMACEUTICALS CO., LTD | Salviae miltiorrhizae radix et rhizoma; Carthami flos | Sodium danshensu; Protocatechuic aldehyde; 4-Hydroxycinnamic acid; Rosmarinic acid; Salvianolic acid B | 10ml/Ampullen;  6Ampullen/box | Y-Prepared according to Chinese pharmacopeia(2010) | Y-HPLC |
| Li et al.(2009) | Danhong injection(DH) | Not mentioned | Salviae miltiorrhizae radix et rhizoma; Carthami flos | / | / | / | / |
| He et al.(2014) | Danhong injection(DH) | SHANDONG BUCHANG PHARMACEUTICALS CO., LTD | Salviae miltiorrhizae radix et rhizoma; Carthami flos | Sodium danshensu; Protocatechuic aldehyde; 4-Hydroxycinnamic acid; Rosmarinic acid; Salvianolic acid B | 10ml/Ampullen;  6Ampullen/box | Y-Prepared according to Chinese pharmacopeia(2010) | Y-HPLC |
| Sui et al.(2014) | Danhong injection(DH) | Not mentioned | Salviae miltiorrhizae radix et rhizoma; Carthami flos | / | / | / | / |
| Wang et al.(2012) | Danhong injection(DH) | Not mentioned | Salviae miltiorrhizae radix et rhizoma; Carthami flos | / | / | / | / |
| Hu et al.(2013) | Danhong injection(DH) | Not mentioned | Salviae miltiorrhizae radix et rhizoma; Carthami flos | / | / | / | / |
| Chen et al.(2013) | Danhong injection(DH) | Not mentioned | Salviae miltiorrhizae radix et rhizoma; Carthami flos | / | / | / | / |
| An et al.(2014) | Danhong injection(DH) | Not mentioned | Salviae miltiorrhizae radix et rhizoma; Carthami flos | / | / | / | / |
| Wang et al.(2015) | Danhong injection(DH) | SHANDONG BUCHANG PHARMACEUTICALS CO., LTD | Salviae miltiorrhizae radix et rhizoma; Carthami flos | Sodium danshensu; Protocatechuic aldehyde; 4-Hydroxycinnamic acid; Rosmarinic acid; Salvianolic acid B | 10ml/Ampullen;  6Ampullen/box | Y-Prepared according to Chinese pharmacopeia(2010) | Y-HPLC |
| Ren et al.(2011) | Danhong injection(DH) | SHANDONG BUCHANG PHARMACEUTICALS CO., LTD | Salviae miltiorrhizae radix et rhizoma; Carthami flos | Sodium danshensu; Protocatechuic aldehyde; 4-Hydroxycinnamic acid; Rosmarinic acid; Salvianolic acid B | 10ml/Ampullen;  6Ampullen/box | Y-Prepared according to Chinese pharmacopeia(2010) | Y-HPLC |
| Lve et al.(2012) | Danhong injection(DH) | Not mentioned | Salviae miltiorrhizae radix et rhizoma; Carthami flos | / | / | / | / |
| Xie et al.(2016) | Danhong injection(DH) | Not mentioned | Salviae miltiorrhizae radix et rhizoma; Carthami flos | / | / | / | / |
| Ye et al.(2012) | Danhong injection(DH) | Not mentioned | Salviae miltiorrhizae radix et rhizoma; Carthami flos | / | / | / | / |
| Cai et al.(2010) | Danhong injection(DH) | SHANDONG BUCHANG PHARMACEUTICALS CO., LTD | Salviae miltiorrhizae radix et rhizoma; Carthami flos | Sodium danshensu; Protocatechuic aldehyde; 4-Hydroxycinnamic acid; Rosmarinic acid; Salvianolic acid B | 10ml/Ampullen;  6Ampullen/box | Y-Prepared according to Chinese pharmacopeia(2010) | Y-HPLC |
| Yang et al.(2016) | Danhong injection(DH) | SHANDONG BUCHANG PHARMACEUTICALS CO., LTD | Salviae miltiorrhizae radix et rhizoma; Carthami flos | Sodium danshensu; Protocatechuic aldehyde; 4-Hydroxycinnamic acid; Rosmarinic acid; Salvianolic acid B | 10ml/Ampullen;  6Ampullen/box | Y-Prepared according to Chinese pharmacopeia(2010) | Y-HPLC |
| Yang et al.(2012) | Danhong injection(DH) | SHANDONG BUCHANG PHARMACEUTICALS CO., LTD | Salviae miltiorrhizae radix et rhizoma; Carthami flos | Sodium danshensu; Protocatechuic aldehyde; 4-Hydroxycinnamic acid; Rosmarinic acid; Salvianolic acid B | 10ml/Ampullen;  6Ampullen/box | Y-Prepared according to Chinese pharmacopeia(2010) | Y-HPLC |
| Deng et al.(2012) | Danhong injection(DH) | SHANDONG BUCHANG PHARMACEUTICALS CO., LTD | Salviae miltiorrhizae radix et rhizoma; Carthami flos | Sodium danshensu; Protocatechuic aldehyde; 4-Hydroxycinnamic acid; Rosmarinic acid; Salvianolic acid B | 10ml/Ampullen;  6Ampullen/box | Y-Prepared according to Chinese pharmacopeia(2010) | Y-HPLC |
| Feng et al.(2013) | Danhong injection(DH) | SHANDONG BUCHANG PHARMACEUTICALS CO., LTD | Salviae miltiorrhizae radix et rhizoma; Carthami flos | Sodium danshensu; Protocatechuic aldehyde; 4-Hydroxycinnamic acid; Rosmarinic acid; Salvianolic acid B | 10ml/Ampullen;  6Ampullen/box | Y-Prepared according to Chinese pharmacopeia(2010) | Y-HPLC |
| Gong et al.(2010) | Danhong injection(DH) | SHANDONG BUCHANG PHARMACEUTICALS CO., LTD | Salviae miltiorrhizae radix et rhizoma; Carthami flos | Sodium danshensu; Protocatechuic aldehyde; 4-Hydroxycinnamic acid; Rosmarinic acid; Salvianolic acid B | 10ml/Ampullen;  6Ampullen/box | Y-Prepared according to Chinese pharmacopeia(2010) | Y-HPLC |
| Guo et al.(2008) | Danhong injection(DH) | Not mentioned | Salviae miltiorrhizae radix et rhizoma; Carthami flos | / | / | / | / |
| Liao et al.(2014) | Dengzhanhua injection(DZHS) | Kunming Longjin Pharmaceutical Co.,LTD. | Erigerontis herba | scutellarin | 10mg/vial; 10vials/box | Y-Prepared according to Chinese pharmacopeia(2010) | Y-HPLC |
| Lan et al.(2007) | Dengzhanhua injection(DZHS) | Not mentioned | Erigerontis herba | / | / | / | / |
| Li et al.(2015) | Dengzhanhua injection(DZHS) | Not mentioned | Erigerontis herba | / | / | / | / |
| Jin et al.(2010) | Dengzhanhua injection(DZHS) | Not mentioned | Erigerontis herba | / | / | / | / |
| Peng et al.(2012) | Dengzhanhua injection(DZHS) | Kunming Longjin Pharmaceuticalco.,LTD | Erigerontis herba | scutellarin | 50mg/vial; 4vials/box | Y-Prepared according to Chinese pharmacopeia(2010) | Y-HPLC |
| Wang et al.(2009) | Chuanxiongqin injection(CXQ) | Wuxi NO.7 Pharmaceutical Co., LTD. | Chuanxiong rhizoma | Tetramethylpyrazine phosphate | 2ml/Ampullen; 10Ampullen/box | Y-Prepared according to Chinese pharmacopeia(2005) | Y-Spectrophotometry |
| Sun et al.(2007) | Chuanxiongqin injection(CXQ) | Harbin Medisan Pharmaceutical Co., LTD. | Chuanxiong rhizoma | Tetramethylpyrazine phosphate | 40mg/vial; 10vials/box | Y-Prepared according to Chinese pharmacopeia(2005) | Y-Spectrophotometry |
| Yu et al.(2008) | Chuanxiongqin injection(CXQ) | Not mentioned | Chuanxiong rhizoma | / | / | / | / |
| Dong et al.(2018) | Chuanxiongqin injection(CXQ) | Anhui Anke Xinxing Pharmaceutical Co.,LTD. | Chuanxiong rhizoma | Tetramethylpyrazine phosphate | 80mg/vial; 6vials/box | Y-Prepared according to Chinese pharmacopeia(2005) | Y-Spectrophotometry |
| Peng et al.(2011) | Danshenchuanxiongqin injection(DSCXQ) | Not mentioned | Salviae miltiorrhizae radix et rhizoma; Chuanxiong rhizoma | / | / | / | / |
| Li et al.(2011) | Danshenchuanxiongqin injection(DSCXQ) | Guizhou Beite Pharmaceutical Co., LTD. | Salviae miltiorrhizae radix et rhizoma; Chuanxiong rhizoma | Ligustrazine hydrochloride; Danshensu | 5ml/Ampullen;  1Ampullen/box | Y-National Standards for Western Medicine | Y-HPLC |
| Sun et al.(2013) | Danshenchuanxiongqin injection(DSCXQ) | Guizhou Beite Pharmaceutical Co., LTD. | Salviae miltiorrhizae radix et rhizoma; Chuanxiong rhizoma | Ligustrazine hydrochloride; Danshensu | 5ml/Ampullen;  1Ampullen/box | Y-National Standards for Western Medicine | Y-HPLC |
| Xie et al.(2011) | Danshenchuanxiongqin injection(DSCXQ) | Guizhou Beite Pharmaceutical Co., LTD. | Salviae miltiorrhizae radix et rhizoma; Chuanxiong rhizoma | Ligustrazine hydrochloride; Danshensu | 5ml/Ampullen;  1Ampullen/box | Y-National Standards for Western Medicine | Y-HPLC |
| Zhang et al.(2016) | Danshenchuanxiongqin injection(DSCXQ) | Not mentioned | Salviae miltiorrhizae radix et rhizoma; Chuanxiong rhizoma | / | / | / | / |
| Zhao et al.(2021) | Danshenchuanxiongqin injection(DSCXQ) | Guizhou Beite Pharmaceutical Co., LTD. | Salviae miltiorrhizae radix et rhizoma; Chuanxiong rhizoma | Ligustrazine hydrochloride; Danshensu | 5ml/Ampullen;  1Ampullen/box | Y-National Standards for Western Medicine | Y-HPLC |
| Cai et al.(2013) | Yinxingye injection(YXY) | Shanxi Powerdone Pharmaceutics Co., LTD. | Ginkgo folium | Total flavonol glycosides ;Terpene lactone | 10ml/Ampullen; 5 Ampullen/box | Y-Prepared according to Chinese pharmacopeia(2010) | Y-HPLC |
| Zhou et al.(2013) | Yinxingye injection(YXY) | Shanxi Powerdone Pharmaceutics Co., LTD. | Ginkgo folium | Total flavonol glycosides ;Terpene lactone | 10ml/Ampullen; 5 Ampullen/box | Y-Prepared according to Chinese pharmacopeia(2010) | Y-HPLC |
| Chen et al.(2011) | Yinxingye injection(YXY) | Shanxi Powerdone Pharmaceutics Co., LTD. | Ginkgo folium | Total flavonol glycosides ;Terpene lactone | 10ml/Ampullen; 5 Ampullen/box | Y-Prepared according to Chinese pharmacopeia(2010) | Y-HPLC |
| Dai et al.(2013) | Yinxingye injection(YXY) | Shanxi Powerdone Pharmaceutics Co., LTD. | Ginkgo folium | Total flavonol glycosides ;Terpene lactone | 10ml/Ampullen; 5 Ampullen/box | Y-Prepared according to Chinese pharmacopeia(2010) | Y-HPLC |
| Cao et al.(2009) | Yinxingye injection(YXY) | Shanxi Powerdone Pharmaceutics Co., LTD. | Ginkgo folium | Total flavonol glycosides ;Terpene lactone | 10ml/Ampullen; 5 Ampullen/box | Y-Prepared according to Chinese pharmacopeia(2010) | Y-HPLC |
| Lu et al.(2015) | Yinxingye injection(YXY) | Shanxi Powerdone Pharmaceutics Co., LTD. | Ginkgo folium | Total flavonol glycosides ;Terpene lactone | 10ml/Ampullen; 5 Ampullen/box | Y-Prepared according to Chinese pharmacopeia(2010) | Y-HPLC |
| Chen et al.(2012) | Yinxingye injection(YXY) | Shanxi Powerdone Pharmaceutics Co., LTD. | Ginkgo folium | Total flavonol glycosides ;Terpene lactone | 10ml/Ampullen; 5 Ampullen/box | Y-Prepared according to Chinese pharmacopeia(2010) | Y-HPLC |
| Li et al.(2010) | Yinxingye injection(YXY) | Shanxi Powerdone Pharmaceutics Co., LTD. | Ginkgo folium | Total flavonol glycosides ;Terpene lactone | 10ml/Ampullen; 5 Ampullen/box | Y-Prepared according to Chinese pharmacopeia(2010) | Y-HPLC |
| Jiang et al.(2009) | Yinxingye injection(YXY) | Shanxi Powerdone Pharmaceutics Co., LTD. | Ginkgo folium | Total flavonol glycosides ;Terpene lactone | 10ml/Ampullen; 5 Ampullen/box | Y-Prepared according to Chinese pharmacopeia(2010) | Y-HPLC |
| Li et al.(2017) | Yinxingye injection(YXY) | Shanxi Powerdone Pharmaceutics Co., LTD. | Ginkgo folium | Total flavonol glycosides ;Terpene lactone | 10ml/Ampullen; 5 Ampullen/box | Y-Prepared according to Chinese pharmacopeia(2010) | Y-HPLC |
| Bao et al.(2010) | Yinxingye injection(YXY) | Shanxi Powerdone Pharmaceutics Co., LTD. | Ginkgo folium | Total flavonol glycosides ;Terpene lactone | 10ml/Ampullen; 5 Ampullen/box | Y-Prepared according to Chinese pharmacopeia(2010) | Y-HPLC |
| Wang et al.(2012) | Yinxingye injection(YXY) | Shanxi Powerdone Pharmaceutics Co., LTD. | Ginkgo folium | Total flavonol glycosides ;Terpene lactone | 10ml/Ampullen; 5 Ampullen/box | Y-Prepared according to Chinese pharmacopeia(2010) | Y-HPLC |
| Zhao et al.(2019) | Yinxingye injection(YXY) | Shanxi Powerdone Pharmaceutics Co., LTD. | Ginkgo folium | Total flavonol glycosides ;Terpene lactone | 10ml/Ampullen; 5 Ampullen/box | Y-Prepared according to Chinese pharmacopeia(2010) | Y-HPLC |
| Yang et al.(2011) | Yinxingye injection(YXY) | Shanxi Powerdone Pharmaceutics Co., LTD. | Ginkgo folium | Total flavonol glycosides ;Terpene lactone | 10ml/Ampullen; 5 Ampullen/box | Y-Prepared according to Chinese pharmacopeia(2010) | Y-HPLC |
| Xiao et al.(2010) | Yinxingye injection(YXY) | Shanxi Powerdone Pharmaceutics Co., LTD. | Ginkgo folium | Total flavonol glycosides ;Terpene lactone | 10ml/Ampullen; 5 Ampullen/box | Y-Prepared according to Chinese pharmacopeia(2010) | Y-HPLC |
| Ye et al.(2012) | Yinxingye injection(YXY) | Shanxi Powerdone Pharmaceutics Co., LTD. | Ginkgo folium | Total flavonol glycosides ;Terpene lactone | 10ml/Ampullen; 5 Ampullen/box | Y-Prepared according to Chinese pharmacopeia(2010) | Y-HPLC |
| Gao et al.(2010) | Yinxingye injection(YXY) | Shanxi Powerdone Pharmaceutics Co., LTD. | Ginkgo folium | Total flavonol glycosides ;Terpene lactone | 10ml/Ampullen; 5 Ampullen/box | Y-Prepared according to Chinese pharmacopeia(2010) | Y-HPLC |
| Wang et al.(2009) | Yinxingye injection(YXY) | Shanxi Powerdone Pharmaceutics Co., LTD. | Ginkgo folium | Total flavonol glycosides ;Terpene lactone | 10ml/Ampullen; 5 Ampullen/box | Y-Prepared according to Chinese pharmacopeia(2010) | Y-HPLC |
| Si et al.(2003) | Gegensu injection(GGS) | Zhejiang Conba Pharmaceutical Co., LTD. | Puerariae lobatae radix | Puerarin | 2ml/Ampullen; 10Ampullen/box | Y-Prepared according to Chinese pharmacopeia(2005) | Y-HPLC |
| Yang et al.(2004) | Gegensu injection(GGS) | Jiangsu Huai'an Shuanghe Pharmaceutical Co., LTD. | Puerariae lobatae radix | Puerarin | 100ml: puerarin 0.2g and glucose 5g | Y-Prepared according to Chinese pharmacopeia(2005) | Y-HPLC |
| Li et al.(2011) | Gegensu injection(GGS) | Not mentioned | Puerariae lobatae radix | / | / | / | / |
| Zhang et al.(2007) | Gegensu injection(GGS) | Beijing 4 ring the PO pharmaceutical Co., LTD. | Puerariae lobatae radix | Puerarin | 0.2g/Ampullen; 4 Ampullen/box | Y-Prepared according to Chinese pharmacopeia(2005) | Y-HPLC |
| Zhang et al.(2007) | Honghua injection(HH) | Not mentioned | Carthami flos | / | / | / | / |
| Liang et al.(2010) | Honghua injection(HH) | Hubei Minkang Pharmaceutical Co., LTD. | Carthami flos | Hydroxysafflor yellow A; Kaempferol | 20ml/Ampullen; 5 Ampullen/box | Y-Prepared according to Chinese pharmacopeia(2010) | Y-HPLC |
| Huang et al.(2006) | Honghua injection(HH) | Sanjiu (Rongcheng) Marine Biotechnology Co., LTD. | Carthami flos | Hydroxysafflor yellow A; Kaempferol | 5ml/Ampullen; 4 Ampullen/box | Y-Prepared according to Chinese pharmacopeia(2010) | Y-HPLC |
| Peng et al.(2011) | Kudiezi injection(KDZ) | Tonghua Huaxia Pharmaceutical Co., LTD. | Thlaspi herba | Total flavonoids; Adenosine | 10ml/Ampullen; 10 Ampullen/box | Y-Compilation of national standards for Chinese medicine | Y-HPLC |
| Teng et al.(2008) | Kudiezi injection(KDZ) | Not mentioned | Thlaspi herba | Total flavonoids; Adenosine | / | / | / |
| Wang et al.(2020) | Kudiezi injection(KDZ) | Tonghua Huaxia Pharmaceutical Co., LTD. | Thlaspi herba | Total flavonoids; Adenosine | 10ml/Ampullen; 10 Ampullen/box | Y-Compilation of national standards for Chinese medicine | Y-HPLC |
