# Supplementary material for: The efficacy and safety of mecobalamin combined with Chinese medicine injections in the treatment of diabetic peripheral neuropathy: A systematic review and Bayesian network meta-analysis of randomized controlled trials
Source: Front Pharmacol. 2022 Nov 4;13:957483. doi: 10.3389/fphar.2022.957483 (PMC9672474; doi:10.3389/fphar.2022.957483)
Supplement: Supplementary file 6 [file DataSheet9.DOCX]

**Supplementary material 12:** Consistency test of common peroneal sensory nerve conduction velocity.

| **Intervention** | **P** | **SD** | **MD(95%CI)** |
| --- | --- | --- | --- |
| ME+CXQ VS ME | 0.34 | 1.9485919 | 2.0(-2.10, 6.1) |
| ME+DH VS ME | ＜0.0001 | 1.9485919 | 5.03(4.018, 6.05) |
| ME+DSCXQ VS ME | ＜0.0001 | 1.9485919 | 4.21(2.39, 6.04) |
| ME+DZHS VS ME | ＜0.0001 | 1.9485919 | 4.03(2.18, 5.87) |
| ME+DZXX VS ME | 0.002 | 1.9485919 | 6.5(2.45, 10.54) |
| ME+GGS VS ME | ＜0.0001 | 1.9485919 | 5.64(3.60, 7.68) |
| ME+HH VS ME | ＜0.0001 | 1.9485919 | 6.25(3.75, 8.75) |
| ME+KDZ VS ME | ＜0.0001 | 1.9485919 | 5.90(3.61, 8.18) |
| ME+YXY VS ME | ＜0.0001 | 1.9485919 | 4.50(3.17, 5.82) |
