# Supplementary material for: The efficacy and safety of mecobalamin combined with Chinese medicine injections in the treatment of diabetic peripheral neuropathy: A systematic review and Bayesian network meta-analysis of randomized controlled trials
Source: Front Pharmacol. 2022 Nov 4;13:957483. doi: 10.3389/fphar.2022.957483 (PMC9672474; doi:10.3389/fphar.2022.957483)
Supplement: Supplementary file 7 [file DataSheet5.DOCX]

**SUPPLEMENTARY MATERIAL 8:** Consistency test of overall response rate.

| **Intervention** | **P** | **SD** | **RR(95%CI)** |
| --- | --- | --- | --- |
| ME+CXQ VS ME | 0.003 | 0.05512923 | 0.177(0.0597, 0.2951) |
| ME+DH VS ME | ＜0.0001 | 0.05512923 | 0.248(0.2024, 0.2942) |
| ME+DSCXQ VS ME | ＜0.0001 | 0.05512923 | 0.292(0.2000, 0.3857) |
| ME+DZHS VS ME | ＜0.0001 | 0.05512923 | 0.253(0.1385, 0.3678) |
| ME+DZXX VS ME | ＜0.0001 | 0.05512923 | 0.492(0.224, 0.7635) |
| ME+GGS VS ME | ＜0.0001 | 0.05512923 | 0.242(0.1175, 0.3674) |
| ME+HH VS ME | ＜0.0001 | 0.05512923 | 0.363(0.1790, 0.5486) |
| ME+KDZ VS ME | 0.038 | 0.05512923 | 0.113(0.0063, 0.2193) |
| ME+YXY VS ME | ＜0.0001 | 0.05512923 | 0.258(0.1959, 0.3204) |
