# Supplementary material for: The efficacy and safety of mecobalamin combined with Chinese medicine injections in the treatment of diabetic peripheral neuropathy: A systematic review and Bayesian network meta-analysis of randomized controlled trials
Source: Front Pharmacol. 2022 Nov 4;13:957483. doi: 10.3389/fphar.2022.957483 (PMC9672474; doi:10.3389/fphar.2022.957483)
Supplement: Supplementary file 8 [file DataSheet1.PDF]

知网（CNKI）：TKA=(‘糖尿病周围神经病变’+’糖尿病末梢神经病变’+’糖尿病性周围神经病变’+’糖尿病性周围神经病’+’糖尿病外周神经病变’+’糖尿病周围神经性病变’+’糖尿病周围神经病’+’糖尿病周围病变’) and TKA=‘注射液’ and FT=’随机’ 1344 篇

万方（WangFang）：主题:(糖尿病周围神经病变 or 糖尿病末梢神经病变 or 糖尿病性周围神经病变 or 糖尿病性周围神经病 or 糖尿病外周神经病变 or 糖尿病周围神经性病变 or 糖尿病周围神经病 or 糖尿病周围病变) and 主题:(注射液) and 主题:(随机 or RCT) 1363 篇

Sinomed（CBM）：(注射液) AND ("随机"[摘要:智能] OR "RCT"[摘要:智能]) AND ("糖尿病周围神经病变"[常用字段:智能] OR "糖尿病末梢神经病变"[常用字段:智能] OR "糖尿病性周围神经病变"[常用字段:智能] OR "糖尿病性周围神经病"[常用字段:智能] OR "糖尿病外周神经病变"[常用字段:智能] OR "糖尿病周围神经性病变"[常用字段:智能] OR "糖尿病周围神经病"[常用字段:智能] OR "糖尿病周围病变"[常用字段:智能]) 426 篇

WeiPu: (糖尿病周围神经病变+糖尿病末梢神经病变+糖尿病性周围神经病变+糖尿病性周围神经病+糖尿病外周神经病变+糖尿病周围神经性病变+糖尿病周围神经病+糖尿病周围病变) and (注射液)and (随机)

Pubmed :

Search number	Query
6	((("Injections"[Mesh]) OR ((((Injections[Title/Abstract]) OR (Injection*[Title/Abstract])) OR (Injectables[Title/Abstract])) OR (Injectable[Title/Abstract]))) AND (Diabetic peripheral neuropathy[Title/Abstract])) AN
5	(RCT[Title/Abstract]) OR (Random*[Title/Abstract])
4	Diabetic peripheral neuropathy[Title/Abstract]
3	("Injections"[Mesh]) OR ((((Injections[Title/Abstract]) OR (Injection*[Title/Abstract])) OR (Injectables[Title/Abstract])) OR (Injectable[Title/Abstract]))
2	(((Injections[Title/Abstract]) OR (Injection*[Title/Abstract])) OR (Injectables[Title/Abstract])) OR (Injectable[Title/Abstract])
1	"Injections"[Mesh]

Embase：

#1 ‘Diabetic peripheral neuropathy’:ab,ti, OR ‘peripheral neuropathy’:ab,ti

#2 Random*:ab,ti

#3 ‘injection’:ab,ti, OR ‘injections’:ab,ti

#4 #1 AND #2 AND #3

170 篇

WOS:

#1 Diabetic peripheral neuropathy OR peripheral neuropathy

#2 Random*

#3 injection or injections or injectable or injectables

#4 #1 AND #2 AND #3

170 篇

Cochrane:

#1 MeSH descriptor: [injections] explode all trees

#2 (Injection*):ti,ab,kw OR (Injections) :ti,ab,kw OR (injectable) :ti,ab,kw OR (injectables) :ti,ab,kw

#3 #1 AND #2

#4 (Diabetic peripheral neuropathy):ti,ab,kw

#5 (Random*):ti,ab,kw

#6 #3 AND #4 AND #5

#1	MeSH descriptor: [Injections] explode all trees	MeSH	23304
#2	(Injection*):ti,ab,kw OR (Injections):ti,ab,kw OR (Injectables):ti,ab,kw OR (Injectable):ti,ab,kw	Limits	103195
#3	#1 or #2	Limits	103249
#4	(Diabetic peripheral neuropathy):ti,ab,kw	Limits	1996
#5	(Random*):ti,ab,kw	Limits	1143429
#6	#3 and #4 and #5	Limits	76

76 篇
