# Supplementary material for: The efficacy and safety of mecobalamin combined with Chinese medicine injections in the treatment of diabetic peripheral neuropathy: A systematic review and Bayesian network meta-analysis of randomized controlled trials
Source: Front Pharmacol. 2022 Nov 4;13:957483. doi: 10.3389/fphar.2022.957483 (PMC9672474; doi:10.3389/fphar.2022.957483)
Supplement: Supplementary file 9 [file DataSheet8.DOCX]

**Supplementary material 11:** Consistency test of common peroneal motor nerve conduction velocity.

| **Intervention** | **P** | **SD** | **MD(95%CI)** |
| --- | --- | --- | --- |
| ME+CXQ VS ME | 0.01 | 1.9855847 | 5.5(1.28, 9.71) |
| ME+DH VS ME | ＜0.0001 | 1.9855847 | 4.73(3.67, 5.80) |
| ME+DSCXQ VS ME | ＜0.0001 | 1.9855847 | 4.60(2.66, 6.55) |
| ME+DZHS VS ME | ＜0.0001 | 1.9855847 | 5.58(3.43, 7.72) |
| ME+DZXX VS ME | 0.003 | 1.9855847 | 6.35(2.1, 10.60) |
| ME+GGS VS ME | ＜0.0001 | 1.9855847 | 6.10(4.03, 8.17) |
| ME+HH VS ME | ＜0.0001 | 1.9855847 | 6.80(4.25, 9.36) |
| ME+KDZ VS ME | ＜0.0001 | 1.9855847 | 5.50(3.20, 7.80) |
| ME+YXY VS ME | ＜0.0001 | 1.9855847 | 5.28(3.89, 6.67) |
